# Receptor-activated transcription factors and beyond: multiple modes of Smad2/3-dependent transmission of TGF-β signaling

**DOI:** 10.1016/j.jbc.2024.107256

**Published:** 2024-04-02

**Authors:** Keiji Miyazawa, Yuka Itoh, Hao Fu, Kohei Miyazono

**Affiliations:** 1 Department of Biochemistry, Graduate School of Medicine, University of Yamanashi, Yamanashi, Japan; 2 Department of Applied Pathology, Graduate School of Medicine, The University of Tokyo, Tokyo, Japan; 3 Laboratory for Cancer Invasion and Metastasis, RIKEN Center for Integrative Medical Sciences, Yokohama, Japan

**Keywords:** TGF-β, Smad, signal transduction, transcription, phosphorylation

## Abstract

Transforming growth factor β (TGF-β) is a pleiotropic cytokine that is widely distributed throughout the body. Its receptor proteins, TGF-β type I and type II receptors, are also ubiquitously expressed. Therefore, the regulation of various signaling outputs in a context-dependent manner is a critical issue in this field. Smad proteins were originally identified as signal-activated transcription factors similar to signal transducer and activator of transcription proteins. Smads are activated by serine phosphorylation mediated by intrinsic receptor dual specificity kinases of the TGF-β family, indicating that Smads are receptor-restricted effector molecules downstream of ligands of the TGF-β family. Smad proteins have other functions in addition to transcriptional regulation, including post-transcriptional regulation of micro-RNA processing, pre-mRNA splicing, and m^6^A methylation. Recent technical advances have identified a novel landscape of Smad-dependent signal transduction, including regulation of mitochondrial function without involving regulation of gene expression. Therefore, Smad proteins are receptor-activated transcription factors and also act as intracellular signaling modulators with multiple modes of function. In this review, we discuss the role of Smad proteins as receptor-activated transcription factors and beyond. We also describe the functional differences between Smad2 and Smad3, two receptor-activated Smad proteins downstream of TGF-β, activin, myostatin, growth and differentiation factor (GDF) 11, and Nodal.

## Smad2/3 as signaling mediators: evolutionary aspects

**Figure 1:**
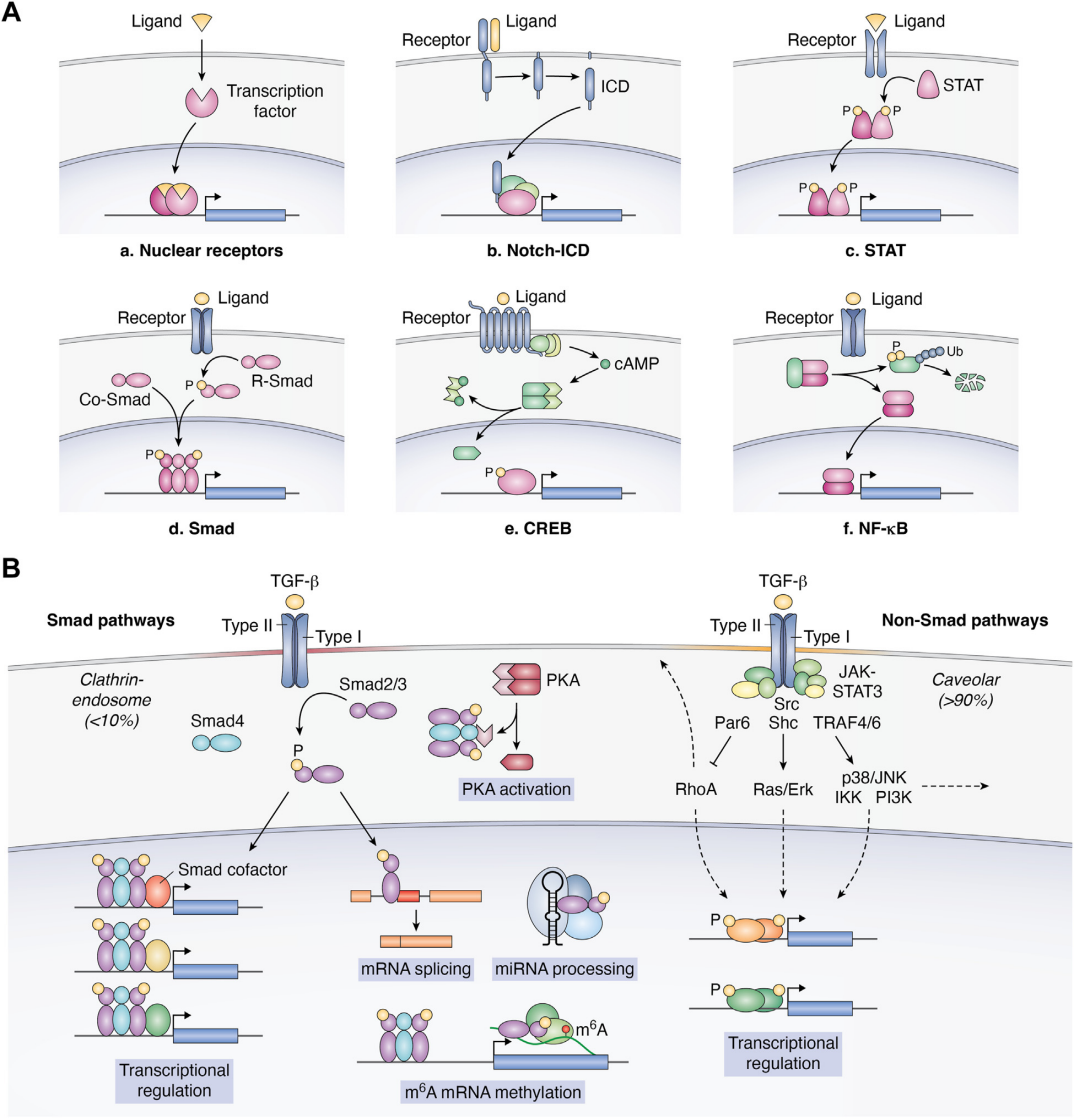
**TGF-β and the Smad signaling system.***A*, modalities of signaling-regulated transcription (1). (*a*) Nuclear receptors are transcription factors activated by membrane-permeable compounds. (*b*) The Notch intracellular domain (ICD) is released by intramembrane proteolysis upon activation of Notch receptors, and translocates into the nucleus to regulate gene expression together with other transcription factors. (*c*) STAT proteins are phosphorylated by receptor-associated tyrosine kinases (JAKs) or other receptor tyrosine kinases, form dimeric complexes, and regulate gene expression in the nucleus. (*d*) Receptor-regulated Smads are serine-phosphorylated by receptor dual kinases, form heterotrimeric complexes with Co-Smad, translocate into the nucleus, and regulate gene expression. (*e*) CREB is located in the nucleus and phosphorylated by PKA. PKA is transiently activated by cAMP. The G-protein–coupled receptor signaling system has two steps for signaling amplification (activation of heterotrimeric G-proteins and the second messenger cAMP production). (*f*) NF-κB is sequestered by IκB in the cytoplasm in a resting state. Signaling-induced IκB phosphorylation triggers its ubiquitylation and subsequent proteasomal degradation, resulting in the release of NF-κB, which translocates into the nucleus to regulate gene expression. *B*, TGF-β transmits signals *via* Smad and non-Smad pathways. In Smad pathways (*left*), Smad2/3 activated by type I receptors upon ligand stimulation form a heteromeric complex with Smad4, regulating gene expression together with other transcription factors (Smad cofactors) in the nucleus. The activated Smad proteins also modulate miRNA processing, mRNA splicing, or m^6^A mRNA methylation. Alternatively, the Smad3–Smad4 complex activates PKA independently of cAMP. Non-Smad pathways (*right*) are transmitted from the caveolar compartment and utilize commonly shared signaling molecules including TRAF4/6, Shc, Src, Par6, and JAK-STAT3.

Intercellular signaling molecules play crucial roles in embryonic development and adult homeostasis in multicellular organisms. The physical interaction of ligands with cognate receptors generates a signal that is transduced to intracellular biochemical reactions, leading to transcriptional regulation of target genes and post-translational regulation of cytosolic or membrane proteins, thus regulating cellular functions. However, the signal transmission modalities vary among signaling pathways (Fig. 1*A*) (1).

Proteins of the metazoan nuclear receptor family receive signals in the form of chemical compounds that cross the plasma membrane from extracellular to intracellular spaces. These receptors are activated in the cytosol or nucleus and subsequently recruit coactivators to specific genomic regions to regulate gene expression. To expand the usage of ligands that cannot cross the plasma membrane, the Notch signaling system adopted receptors that interact with signaling molecules at the plasma membrane. The Notch membrane receptors release their intracellular domain through intramembrane proteolysis upon activation by ligands, and the intracellular domains translocate into the nucleus, where they regulate gene expression in cooperation with other DNA-binding transcription factors. Similar to nuclear receptors, however, the Notch system is devoid of signal amplification processes.

In subsequent evolutionary steps, signal amplification processes mediated by post-translational modifications other than intramembrane proteolysis were introduced. One such amplification process is the activation of transcription factors by reversible phosphorylation. Many cytokines and growth factors that activate the signal transducer and activator of the transcription (STAT) system and the Smad system follow this mode of activation. In these cases, cytosolic signaling molecules are directly phosphorylated by activated receptor complexes, followed by translocation into the nucleus. STAT proteins are activated by many cytokine receptors, including TGF-β receptors, *via* the receptor-associated tyrosine kinase JAK or receptor tyrosine kinases. By contrast, Smad proteins are activated exclusively by intrinsic receptor serine/threonine kinases of the TGF-β family, indicating that Smad proteins are receptor-restricted effector molecules. In other cases, the amplification process is inserted upstream of transcription factor–activating kinases. G-protein coupled receptors utilize the second messenger cAMP for signaling amplification and activate protein kinase A, which phosphorylates nuclear cAMP response element binding protein (CREB). Many growth factors and cytokines that signal *via* tyrosine phosphorylation utilize the mitogen-activated protein kinase (MAPK) cascade for amplification. The cascade includes extracellular signal–regulated kinase (ERK), c-Jun N-terminal kinase (JNK), p38 MAPK, and upstream kinases and mediates the phosphorylation and activation of various transcription factors including the Ets, Jun, and ATF family proteins in the nucleus. In these cases, transcription factor–activating kinases translocate into the nucleus. Kinases that directly phosphorylate transcription factors in the former systems are tethered to the plasma membrane, whereas those in the latter system are amplified and more motile in the intracellular space. Therefore, the STAT and Smad systems appear to be evolutionary closer to the prototypic Notch system.

Innate immune signaling pathways are activated by the recognition of pathogen-associated molecular patterns by pattern-recognition receptors and have unique modes of signal transmission. Receptors in this pathway are localized at the plasma membrane or cytosol. Protein kinases that phosphorylate and activate downstream transcription factors (the IRF family of transcription factors) are cytosolic enzymes and can receive signals either from membrane receptors or cytosolic receptors *via* adaptor molecules such as MAVS, STING, and TRIF (2).

Other amplification systems involve de-repression of transcription factors accompanying proteolysis. β-catenin, a downstream transcription factor of Wnt signaling, is constitutively degraded by phosphorylation-triggered ubiquitylation, followed by proteasomal degradation. Activation of the Wnt pathway suppresses the degradation and stabilizes β-catenin, which undergoes nuclear translocation to regulate gene expression together with other transcription factors. Gli transcription factors are downstream effectors of the hedgehog signaling pathway. In the resting state, Gli proteins are sequestered in the cytoplasm by the suppressor protein SuFu, which results in their inactivation, or processed into repressor forms by proteolytic cleavage to release the C-terminal transactivation domain. Input from hedgehog signaling results in the dissociation of SuFu or blockade of the proteolytic cleavage, which causes the translocation of full-length active Gli proteins into the nucleus. Activation of the NF-κB pathway involves phosphorylation-triggered proteasomal degradation of IκB, a negative regulator of NF-κB that sequesters NF-κB in the cytoplasm, allowing NF-κB to translocate into the nucleus.

The Notch pathway is a highly receptor-restricted pathway; the Smad pathway is also receptor-restricted, although to a lesser extent, whereas the STAT pathway is moderately receptor-restricted. Other signaling receptors, tyrosine kinase receptors, and G-protein coupled receptors regulate gene expression *via* commonly shared transcription factors that are activated or repressed by downstream kinases including MAPKs, PKA, PKB, and PKC. Although Smad proteins are essentially receptor-activated transcription factors (3), multiple modes of action have recently been elucidated. Here, we review the conventional as well as newly unveiled roles of the Smad signaling system.

## Smad-dependent and non-Smad pathways in TGF-β signaling

Smad proteins that mediate signaling by TGF-β family members are divided into three classes (4). Smad proteins that are phosphorylated and activated by receptors are termed receptor-regulated Smads (R-Smads) and are further divided into two subgroups: Smad2 and Smad3, which mediate signaling by TGF-β, activins, myostatin, GDF-11, and Nodal; and Smad1, Smad5, and Smad8, which primarily mediate signaling by the bone morphogenetic protein (BMP) subfamily. Smad4 is the only common partner Smad (Co-Smad) in mammals and cooperates with R-Smads to regulate gene expression. Smad6 and Smad7 are induced by TGF-β as well as other signaling pathways and function as negative regulators, and they are therefore termed inhibitory Smads (I-Smads).

The activity of R-Smad proteins as transcriptional regulators is induced by type I receptor-serine/threonine kinases of the TGF-β family (Fig. 1*B*). Upon TGF-β stimulation, TGF-β type I receptor (also known as activin receptor-like kinase 5, ALK-5) and type II receptor form a tetraheteromeric complex in which the type I receptor is phosphorylated and activated by the type II receptor. The activated type I receptor in turn phosphorylates Smad2 or Smad3 at the two distal serine residues in the C-terminal SSXS motif. The phosphorylated Smad2/3 then form heterotrimeric complexes with Smad4 and translocate to the nucleus to regulate target gene expression. As with many other transcription factors, the activated Smad complexes do not form a complete transcriptional unit, but a half unit (5). Therefore, cooperation with other transcription factors is usually required. Transcription factors that cooperate with Smad proteins are collectively termed “Smad cofactors” and will be discussed in detail below. In addition to the TGF-β type I receptor, Smad2/3 are phosphorylated by activin receptor type IB (ALK-4), which is activated by activins, and activin receptor type IC (ALK-7), which is activated by Nodal, a key player in embryonic development.

Non-Smad pathways utilize effectors that are shared with other signaling molecules because TGF-β receptor type I and type II kinases are dual specificity kinases that can also signal *via* tyrosine phosphorylation and the type I receptor can be connected to TRAFs (TNF receptor-associated factors) (6). Non-Smad pathways include the ERK1/2, JNK, p38 MAPK, phosphoinositide 3-kinase (PI3K)/protein kinase B (Akt), STAT3, and c-Src pathways (7, 8, 9, 10, 11, 12, 13). These pathways signal from the caveolar compartment (14), whereas Smad signals are transmitted from the plasma membrane or early endosomes (15). The intensity of non-Smad signaling is generally lower than that of tyrosine kinase receptors (16), which may be due to the low level of cell surface expression of TGF-β receptors. The ERK pathway is triggered by the activated TGF-β type I receptor through tyrosine phosphorylation of the adaptor protein Shc, resulting in docking of the Grb2–Sos1 complex, which activates the Ras-Raf-MEK-ERK pathway (7). Activation of JNK, p38 MAPK, and PI3K is dependent on TRAF4/6 (8, 9, 10, 11). STAT3 is activated by JAK1, which is constitutively associated with the TGF-β type I receptor (12). The c-Src pathway is activated by TGF-β–dependent tyrosine phosphorylation of the type I receptor by the type II receptor (13). The activation of non-Smad pathways appears to be context-dependent. Some pathways known to be “non-Smad” may become Smad-dependent in the late phase after TGF-β stimulation, possibly due to induction of growth factors or downstream signaling molecules by TGF-β. In A549 lung adenocarcinoma cells, activation of the Erk and p38 MAPK pathways occurs independently from Smad3 at 1 h after stimulation, whereas it is dependent on Smad3 at 4 h (Erk) or 16 h (p38 MAPK) after stimulation (17). Delayed-phase STAT3 phosphorylation (later than 2 h after stimulation) is dependent on Smad3 (12). c-Src transcriptionally induced by Smad signaling plays a role in the late phase of activation (18). Because many cell responses induced by TGF-β are Smad dependent, the involvement of non-Smad pathways in the regulation of cellular functions is not well understood. Non-Smad pathways are often indispensable for, but might have supportive effects on, cell responses induced by TGF-β. Therefore, the TGF-β signaling system is a hybrid pathway that includes receptor-restricted effectors and widely used downstream effectors.

## Structure and activation of Smad proteins

### Domain structure of Smad proteins

**Figure 2:**
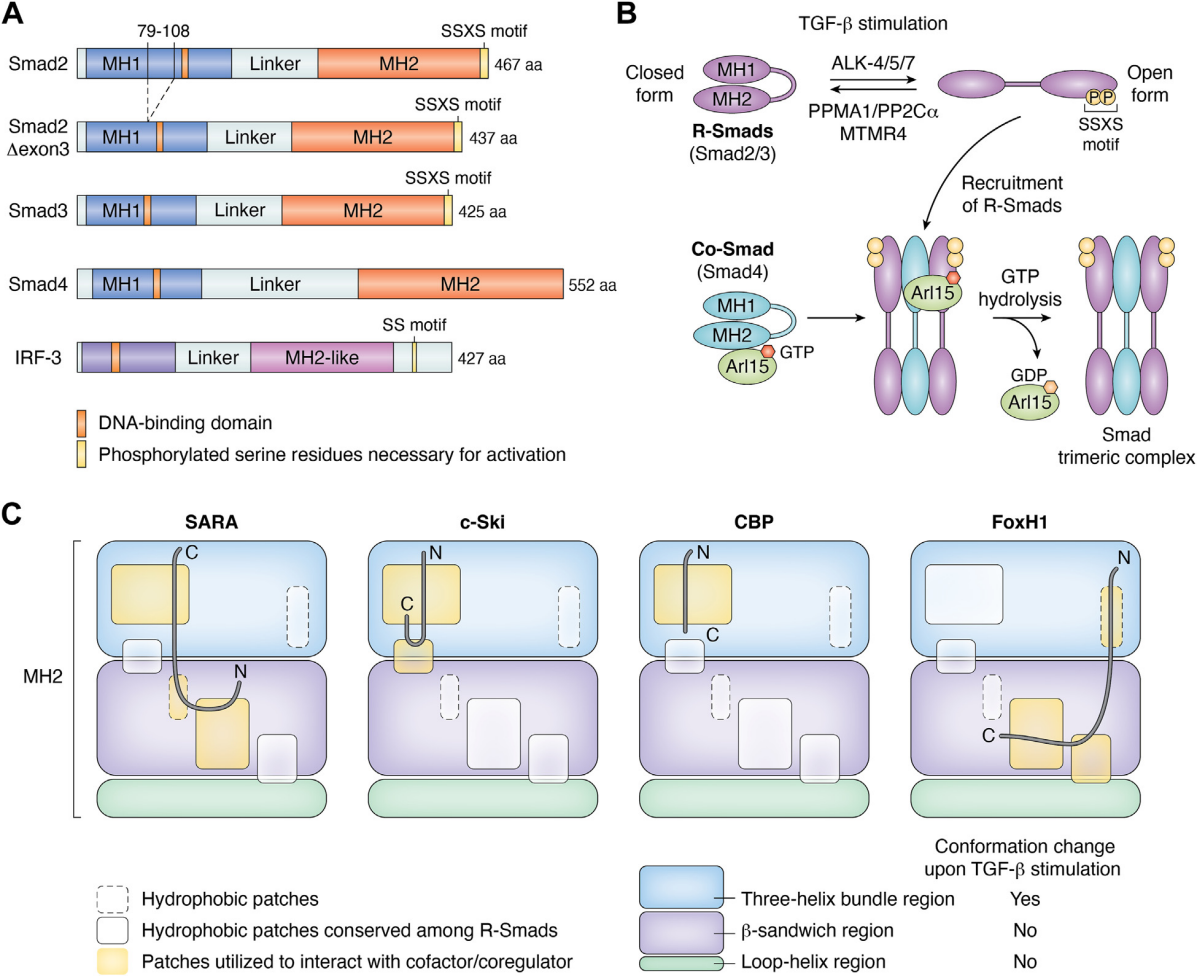
**Structure and activation of Smad proteins.***A*, schematic structures of Smad2, Smad2Δexon3, Smad3, and Smad4. The structure of IRF-3 is shown for comparison. The MH1 domain and MH2 domain are shown in *blue* and *red*, respectively. Phosphorylated serine residues necessary for activation are shown in *yellow*. *B*, activation of Smad proteins. R-Smads (Smad2/3) are in equilibrium between a closed (*left*) and an open (*right*) form. R-Smads favor the open form upon C-terminal phosphorylation by type I receptors, which can be reversed by the protein phosphatase PPMA1/PP2Cα or MTMR4. Co-Smad (Smad4) in its closed form is converted to an open form via interaction with GTP-bound Arl15, depending on TGF-β stimulation (48). R-Smads and Co-Smad in the open conformation form a trimeric complex (the activated Smad complex), in which Co-Smad accelerates GTP hydrolysis by Arl15, resulting in its dissociation from the complex. *C*, Smad-binding proteins utilize a combination of hydrophobic patches in the MH2 domain (110, 111, 112). Interaction of Smad-binding regions of SARA, c-Ski, CBP, and FoxH1 (*gray lines*) with hydrophobic patches is illustrated. Hydrophobic patches are shown as boxes. Those conserved among R-Smads are shown as solid boxes. Those used for interaction with each Smad-binding peptide are shown in *orange*. The three-helix bundle region, β-sandwich region, and loop helix regions are also shown.

R-Smad and Co-Smad share conserved domain structures, in which the N-terminal Mad homology 1 (MH1) and the C-terminal Mad homology 2 (MH2) domains are connected by an intrinsically disordered linker region (Fig. 2*A*). The amino acid sequences of the MH1 and MH2 domains are well conserved, but those of the linker regions are variable. To function as signaling-activated transcription factors, Smad2/3 should contain four functional regions: a region that receives upstream signals, a region that enables nuclear translocation, a DNA-binding region, and a transactivation region. These regions are separately distributed in the structure of Smad proteins.

The MH1 domains of R-Smads and Co-Smad share a well-conserved β-hairpin structure that is involved in DNA binding (19, 20, 21, 22, 23). However, Smad2 does not interact with DNA because exon 3, located N-terminally adjacent to the β-hairpin, interferes with DNA binding. Consistently, an alternatively spliced isoform of Smad2 lacking exon 3, Smad2Δexon3 (also termed Smad2β), can interact with DNA. Moreover, the presence of exon 3 considerably affects the molecular properties of Smad2 such as intracellular localization, oligomeric properties, and interaction with binding partners (24, 25, 26). In *Drosophila melanogaster*, only one Smad2/3 homolog, dSmad2, also termed Smox, has been identified. dSmad2 contains an insert that corresponds to exon three in mammalian Smad2 (27), suggesting that R-Smad lacking DNA-binding properties might be an ancestral form. In the *Xenopus* animal cap assay, *Xenopus* Smad2Δexon3 partially loses the unique *Xenopus* Smad2 features for target gene expression and behaves more similarly to *Xenopus* Smad3 than *Xenopus* Smad2 (28). Knock-in experiments in mice during early embryonic development indicate that Smad2Δexon3 and Smad3 are functionally interchangeable (29). Consistently, Smad2Δexon3 behaves similarly to Smad3 in biochemical assays (30). However, in cell biological assays, Smad2Δexon3 rescues many of the defects resulting from *SMAD3* knockout but fails to rescue cell motility (31).

In addition to the DNA-binding β-hairpin structure, the MH1 domains of Smad3 and Smad4 have nuclear localization signals that contribute to their nuclear transport (32, 33). Some transcription factors that cooperate with Smad complexes, including c-Jun and Sp1, interact *via* the MH1 domain (34, 35).

The MH2 domains of R-Smads contain the L3 loop, a specific structure recognized by type I receptors (36) that ensures the specificity of signal transmission from receptors, and the C-terminal SSXS motif consists of two distal serine residues that are phosphorylated by type I receptors to activate R-Smads as transcription factors. The MH2 domains are also involved in trimeric complex formation, and the resultant trimeric structure serves as an interacting surface for binding proteins including transcription factors, co-activators, co-repressors, and other regulatory molecules. Among the binding proteins that associate with Smad2/3 prior to activation, SARA (Smad anchor for receptor activation) serves to present unphosphorylated Smad2/3 for activation by type I receptors in early endosomes (37, 38, 39); however, this process is not essential for Smad activation (40). The interaction of Smad2/3 with phosphatidylinositol-4,5-bisphosphate (PIP_2_) *via* the MH2 domain was recently reported (41). This interaction mediates the association of Smad proteins with the plasma membrane, which facilitates interaction with type I receptors and therefore C-terminal phosphorylation. Smad C-terminal phosphorylation in turn induces a conformational change of the MH2 domain, which attenuates the interaction between Smad and PIP_2_. The relative contribution of SARA-dependent and PIP_2_-dependent presentations to receptors during the activation of Smad2/3 remains to be evaluated. Nevertheless, the MH2 domain localizes Smad2/3 to the membrane, thereby facilitating phosphorylation by type I receptors. The MH2 domain is also involved in nuclear translocation *via* interaction with nucleoporins (42).

The linker regions have several serine/threonine phosphorylation sites. Although these sites do not directly affect the activation status of R-Smads as transcription factors, they modulate their function by serving as docking sites for interacting proteins (see below). In addition to the MH2 domain, the linker regions of Smad3 and Smad4 are involved in the interaction with p300/CBP and exhibit transactivation activities (43, 44, 45).

### Functional activation of Smad proteins

The mechanism underlying phosphorylation-dependent R-Smad activation was determined soon after the identification of Smad proteins (Fig. 2*B*). R-Smads and a Co-Smad are in equilibrium between open and closed forms through intramolecular interactions between the MH1 and MH2 domains. In the resting unstimulated state, the equilibrium leans toward the autoinhibited closed form, in which each domain is functionally inhibited (46). However, R-Smads in the closed form are not necessarily biologically inactive molecules, and certain functions of unphosphorylated Smad proteins were recently discovered (see below). Missense mutations of conserved arginine residues in the MH1 domains, such as Smad2 (Arg133Cys) and Smad4 (Arg100Thr), have been detected in colon and pancreatic carcinomas, respectively. These mutants enhance the autoinhibitory interaction between the MH1 and MH2 domains, resulting in loss of function in these mutants (46).

Phosphorylation of the C-terminal SSXS motif in the MH2 domain of Smad2/3 induces a conformational change, shifting the equilibrium toward the open form, which favors trimeric complex formation (47). The C-terminal phosphorylation thus drives Smad2 and Smad3 to trimerize with Smad4 through the MH2 domain, which releases the MH1 domain and exposes the DNA binding β-hairpin structure. Therefore, a binding partner of MH2 switches from the intramolecular MH1 domain to the intermolecular MH2 domain, leading to functional activation.

Because Smad4 does not contain the C-terminal SSXS motif, the equilibrium between the inactive closed form and the active open form is not regulated by phosphorylation. Arl15 was recently shown to derepress the autoinhibited Smad4 and facilitate R-Smad–Smad4 complex formation (48) (Fig. 2*B*, lower). Arl15 is a member of the Arf family of small G proteins. GTP-bound Arl15 associates with the Smad4 MH2 domain *via* its own switch-II region (a region that undergoes a conformational change upon GTP binding) and promotes complex formation between Smad4 and R-Smads. Smad4 serves as a low-activity GTPase activating protein for Arl15, but R-Smad binding to Smad4 considerably enhances the GAP activity of Smad4. Therefore, upon formation of the R-Smad–Smad4 trimeric complex, Arl15 is converted into a GDP-bound form and is released from the complex, thereby allowing the translocation of the Smad complex into the nucleus. Arl15 is a palmitoylated protein (49) and localizes to the membrane fraction including endolysosomes (48). Therefore, Arl15 is involved in recruiting Smad4 to membrane fractions where Smad2/3 are phosphorylated by activated TGF-β type I receptors. TGF-β stimulation promotes the interaction between Arl15 and Smad4, indicating that TGF-β signaling is involved in the Arl15-induced activation of Smad4. However, the underlying mechanism remains to be elucidated. It is possible that TGF-β activates guanine nucleotide exchange factors (GEFs) that target Arl15. Analogous cofactors may exist that shift the equilibrium between the inactive and active forms of R-Smads. Although such cofactors have not yet been identified, it is possible that the activity of R-Smad is also regulated by this novel mode of Smad activation.

Smad2/3 mutants thus far identified are mostly loss-of-function mutants. However, gain-of-function mutants of Smad3, namely, Ser264Tyr and Ser264Phe, were identified in endosteal melorheostosis, a disease characterized by mesodermal dysplasia of bone (50). Smad3 Ser-264 mutants are C-terminally phosphorylated in unstimulated cells, and the phosphorylation is not suppressed by inhibition of TGF-β type I receptor kinase. Some kinase(s) other than type I receptors might be involved in this phosphorylation, suggesting another novel mode of Smad activation.

C-terminally phosphorylated active Smad2/3 are deactivated by the phosphatase PPM1A/PP2Cα (51) or MTMR4 (52). PPM1A/PP2Cα is located in the nucleus, and its dephosphorylation activity results in the nuclear export of Smad2/3; MTMR4 contains an FYVE domain and is located in early endosomes, and its dephosphorylation activity suppresses the nuclear translocation of Smad2/3.

### Heterotrimeric Smad complexes

In the open conformation of Smad proteins, both the MH1 and MH2 domains are released from interdomain interaction–mediated autoinhibition. Activated Smad proteins form trimeric structures (53), and the trimeric model was validated by resolution of the crystal structure of the MH2 domains with the C-terminal residues that mimic phosphorylated residues (47, 54). The possible formation of a dimeric Smad complex was also reported (24, 55, 56), and such dimeric complexes may be functional in specific situations (56). However, the structure of a dimeric Smad complex remains to be resolved. The importance of trimeric complex formation is supported by the fact that many pathogenic mutations in Smad 2/3 and Smad4 are located in the trimerization interfaces (57).

To regulate gene expression, Smad complexes need to translocate into the nucleus. This process is mediated by importin- and nucleoporin-dependent mechanisms. The MH1 domains of Smad3 and Smad4 have lysine-rich nuclear localization signals, which mediate the interaction of Smad3 and Smad4 with importin β and importin α, respectively (32, 33). Alternatively, Smad proteins physically interact with nucleoporins *via* the MH2 domain (Smad2 and Smad3) or as a full-length protein (Smad4) (42, 58). Simultaneously, Smad3 and Smad4 acquire DNA-binding ability *via* the β-hairpin region in the MH1 domain.

Three types of heterotrimerization compositions have been suggested: Smad2–Smad2–Smad4, Smad2–Smad3–Smad4, and Smad3–Smad3–Smad4. Although the formation of the Smad2–Smad3–Smad4 complex was detected in TGF-β–stimulated cells using the *in situ* proximity ligation (*in situ* PLA) assay (59), the functional properties of the mixed complexes are not well understood. Recent bioinformatics analyses suggested the existence of Smad complexes with other compositions (60). These complexes, however, have not been experimentally demonstrated to date.

The role of Smad4 in the trimeric complex remains unclear. As will be discussed later, endogenous Smad complexes lacking Smad4 in cultured cells can transmit signals. However, induction or repression of many of the target genes of TGF-β is less effective in the absence of Smad4 (61), suggesting that Smad4 is required for the stable function of activated Smad complexes. Alternatively, because Smad3 and Smad4 have slightly different DNA-binding properties (5), the inclusion of Smad4 provides variation in DNA-binding by activated Smad complexes.

In specialized situations, TGF-β stimulation can induce phosphorylation of Smad1/5 by activin A receptor like type 1 (ALK-1) (62), activin A receptor type 1 (ALK-2) (63), or the TGF-β type I receptor (ALK-5) (64). In such cases, mixed heteromeric complexes composed of Smad2/3 and Smad1/5/8 can be formed (63) and attenuate BMP signaling (65). Similarly, BMP stimulation also induces phosphorylation of Smad2/3 in embryonic cells and transformed cells (66). Therefore, Smad complexes with various compositions can be formed during the transmission of the TGF-β family signals, thereby modulating the signaling output.

Other receptor-activated transcription factors form dimeric complexes upon activation. STAT3 forms a dimer upon tyrosine phosphorylation and dimerizes *via* SH2 domain-mediated mutual interaction (67). IRF-3 forms a functional dimer upon serine/threonine phosphorylation near the C-terminus (Fig. 2*A*), leading to de-repression from autoinhibition by intramolecular interaction (2). Therefore, one characteristic feature of the Smad complex is the formation of trimeric complexes containing Co-Smad.

Although rare, trimeric transcription factors other than the Smad complex have been identified. Heat shock factors (HSFs) are homo/heterotrimeric transcription factors that are activated by stress (68). HSF1 is inactivated in the resting state by an inhibitory intramolecular interaction and activated by external stimuli (stress); it forms homotrimers or heterotrimers with HSF2 and regulates the expression of genes involved in heat shock responses. This mode of activation is reminiscent of that of Smad proteins. However, heat shock elements are usually composed of at least three contiguous inverted repeats of the pentameric motif 5′-nGAAn-3′ (68, 69). This is in contrast to Smad complexes, which usually recognize a solitary 5′-GTCT-3′ or 5′-AGAC-3′ motif.

## Smad proteins as receptor-activated DNA-binding transcription factors: the Smad transcription pathway

To elicit cellular responses to extracellular signaling molecules, signaling-activated transcription factors need to correctly bind to the regulatory regions of target genes *via* specific recognition of DNA elements (70). To ensure the precise recognition of regulatory elements distributed on the genome, transcription factors need to have high specificity and affinity for DNA binding. The monomeric DNA-binding domain of a transcription factor recognizes only a short stretch of a DNA motif with dissociation constants (Kd) in the nanomolar range, which is low considering the concentration of transcription factors in the nucleus. To overcome this, multiple repeats of DNA-binding domains can be present in a single polypeptide, or homomultimeric complexes can be formed. Another way is to form heteromeric complexes with other DNA-binding transcription factors. The latter strategy is used by many eukaryotic transcription factors because the formation of heteromeric complexes enables, *via* combinatorial control, the expansion of the repertoire of binding DNA elements when each component recognizes a different motif or altering the mode of gene regulation by assembling activation domains with different properties.

Smad proteins (except for full-length Smad2) have one DNA binding domain, and even the formation of heterotrimeric complexes by Smad proteins does not improve DNA binding because the occurrence of multiple Smad binding motifs in proximity in the genome is rare. In addition, a single Smad trimeric complex is not sufficient for transcriptional activation, and a Smad trimer is regarded as a transcriptional half unit (5). Therefore, activated Smad proteins have acquired the strategy of forming heteromeric complexes with other DNA-binding transcription factors (collectively termed Smad cofactors). Smad proteins interact with a variety of transcription factors, which confers the versatility of Smad proteins in the context-dependent regulation of target gene expression *via* combinatorial interaction with signaling-activated transcription factors as well as master transcription factors that dictate lineage determination.

One important point is that although Smad2 lacks DNA-binding ability, Smad complexes containing Smad2 still function in transcriptional regulation. Family members or isoforms of transcription factors that lack DNA binding ability act as dominant negative transcription factors upon hetero-oligomerization. Prominent examples include Id proteins, which are helix-loop-helix proteins that lack the DNA-binding basic region and thus inhibit basic helix-loop-helix transcription factors, and C/EBP homologous protein 10 (CHOP), which inhibits DNA-binding of CCAAT/enhancer-binding proteins (C/EBPs) (71). The definite role of Smad2 in transcriptional regulation is not well understood. Knockdown of Smad2 increases the activity of luciferase reporters carrying Smad-binding elements (CAGA-Luc) (72). This probably occurs because these reporters are driven by Smad complexes principally containing Smad3 and Smad4, and Smad2 could function as a partial dominant-negative component. During transcriptional activation of endogenous target genes, the inclusion of Smad2 in the trimeric complex might affect the DNA-binding properties of the activated Smad complexes by replacing Smad3, thus shaping the transcriptional profiles of cellular responses to TGF-β.

### Smad-binding DNA elements

A DNA element that interacts with Smad3 or Smad4 was screened from an oligonucleotide DNA library using bacterially expressed Smad3 or Smad4, and found to be a palindromic sequence of 8-base pairs, 5′-GTCT AGAC-3′ (Smad binding element, SBE) (73). A functional study of a TGF-β–responsive *cis*-element in the promoter region of the human plasminogen activator inhibitor gene (*SERPINE1*) identified the CAGA motif (5′-C/ACAGACA-3′ or its complementary 5′-TGTCTGG/T-3′) (74). Similarly, a functional study of the *JUNB* promoter identified a sequence containing 5′-CAGACA-3′ (75). Crystallographic studies of the DNA-complexed MH1 domain of Smad3 (19, 20) or Smad4 (22) showed that a protruding β-hairpin structure in the MH1 domain is inserted into the major groove of the DNA double helix; specific hydrogen bonds are formed with three bases in the half-site (5′-GTCT-3′) of the SBE motif, where the second T has no contact with Smad proteins. Consistently, a degenerated sequence in the second position of the 5′-GTCT-3′ motif only modestly affected binding to Smad3 (73). These findings demonstrate that Smad proteins interact with DNA essentially *via* 5′-GTCT-3′ or its complementary 5′-AGAC-3′ motif. The Kd for Smad3 and SBE is approximately 100 nM, whereas that for Smad4 and SBE is 200 to 260 nM (19, 76). *In vitro* selection of Smad binding sequences using Smad3 or Smad4 expressed in HEK293T cells with TGF-β signaling input indicated that Smad3 preferentially binds to the CAGA motif (5′-TGTCTGG-3′) over the SBE (5′-GTCTAGAC-3′), whereas Smad4 prefers the SBE to the CAGA motif (5), suggesting that the DNA-binding properties of Smad3 and Smad4 are different.

Smad1 and Smad5, the receptor-regulated Smad proteins that mediate BMP signaling, recognize GC-rich sequences (77, 78). The 5-bp GC-rich sequence 5′-GGCGC-3′ or 5′-GGCCG-3′ binds to Smad3 and Smad4 in addition to Smad1 and Smad5, as revealed by ChIP-seq analysis for Smad2/3 and Smad4 (76). Consistently, a structural study clearly indicated that Smad3 and Smad4 interact with 5-bp GC-rich sequences (76). The Kd for the 5-bp GC-rich motif (5′-GGCGC-3′) and Smad4 is approximately 160 nM, which is slightly lower than the Kd for Smad4 and SBE (200 nM) or the CAGA motif (270 nM). However, the *in vitro* selection of Smad binding sequences described above (5) indicates that 5-bp GC-rich sequences are not preferred by Smad proteins. Although BMP-regulated Smads can activate luciferase reporters with GC-rich sequences (77, 78, 79), a luciferase reporter containing a 5-bp GC-rich sequence that responds to TGF-β has not been developed. Such a reporter would confirm the validity of the model.

ChIP-seq analyses indicate that a composite element (GC-rich SBE, 5′-GGC/AGCC-3′ and 5′-GTCT-3′ motif) is concentrated by BMP-activated Smad complexes and is transcriptionally activated after BMP stimulation (78). By contrast, such robust composite elements are not observed for TGF-β–activated Smad complexes (5, 80, 81, 82). Therefore, the DNA-binding properties of Smad2/3 confer high versatility in the transcriptional regulation of target genes.

### Recruitment of chromatin remodeling enzymes

After DNA-binding transcription factors successfully associate with target gene promoters or enhancers, the transcriptional complex modulates the local chromatin structure by changing the chromatin marks through the recruitment of histone-modifying enzymes to enable gene regulation. Transcription factor-induced alteration in the chromatin structure is required for the activation of transcription at regulatory sites (83).

Coregulators known to interact with Smad2/3 and affect histone acetylation status include coactivators such as p300/CBP (84, 85, 86, 87, 88, 89), p/CAF (90), and RAP250 (91), and corepressors such as TGIF (92), c-Ski (93, 94), SnoN (95), Evi1 (96), and MEL1 (97), which positively and negatively modulate gene expression, respectively.

p300/CBP interacts with Smad2, Smad3, and Smad4 through the MH2 domain (84, 85, 86, 87, 88, 89). However, the linker regions of Smad3 and Smad4 contain additional activation domains (44, 45, 98). In Smad4, this domain is located in a region termed Smad activation domain (SAD), a 48 amino acid proline-rich sequence in the linker region (residues 275–322), through which Smad4 interacts with p300 (43). Smad3 has an additional activation domain at the boundary between the linker and MH2 domain (residues 201–248) that mediates the interaction of Smad3 with p300 or p/CAF (44, 45). Smad3 or Smad4 lacking these regions can form trimeric Smad complexes but cannot activate transcription.

p300 functions as a dimer (99); however, a single Smad trimeric complex is not sufficient for transcriptional activation even if it stably associates with DNA (5). Therefore, two p300 molecules might not simultaneously associate with one Smad trimeric complex, although p300 can interact with both R-Smads and Smad4. The mechanism by which coactivators are incorporated into the activated Smad complexes and successfully contribute to transcriptional activation remains to be elucidated.

In addition to recruiting coactivators or corepressors, activated Smad complexes recruit other histone remodelers to the regulatory sites of target genes. Recruitment of SMARCA4/Brg1, a component of the SWI/SNF chromatin remodeling complex, to the Smad complex, is necessary for directing the basic transcription machinery to transcription start sites, in addition to histone acetyltransferases (100).

Smad2/3 also physically interacts with KDM6B, which demethylates histone H3 K27 trimethylation (H3K27me3), and recruits it to target gene promoter regions to erase the repressive mark, thereby activating gene expression (101, 102). TRIM33, which contains a plant homeodomain (PHD) and a bromodomain, recognizes the repressive histone marks H3K9me3 and H3K18ac. Activated Smad complexes together with TRIM33 (TIF1-γ) are recruited to genomic regions with dual histone marks and de-repress target gene expression by removing heterochromatin protein 1γ (HP1γ) from the region (103). By contrast, Smad3 recruits SETDB1, which catalyzes histone H3 K9 trimethylation (H3K9me3), to the *snail* promoter region and induces its repression *via* the repressive mark (104).

Alteration in epigenetic marks is not limited to histones. Smad-dependent and locus-specific DNA demethylation in response to TGF-β stimulation is observed at the *p15*^*ink4b*^ locus (105). The activated Smad complex recruits a cytidine deaminase, AID, and DNA glycosylases together with the coactivator CBP, displacing a corepressor complex consisting of the DNA binding transcription factor ZNF217, the corepressor CoREST, and the DNA methyltransferase Dnmt3a.

### Smad cofactors

#### Smad2/3–Smad cofactor interaction: *in vitro* context

The interaction of Smad proteins with other transcription factors is thought to underlie context-dependent TGF-β signaling. One important question in the biochemistry of Smad proteins is how such different proteins can specifically interact with Smads to exert their functions. Although the Smad interaction motif (SIM) Pro-Pro-Asn-Lys-Ser is present in FoxH1, Mixer (106), and TMEPAI (107), various proteins that do not share common binding sequences interact with the Smad2/3 MH2 domains (108). These factors interact with shallow hydrophobic regions (hydrophobic patches) on the MH2 domain, sometimes *via* their disordered regions. Based on the interaction between the Smad binding region of SARA and MH2, a model was proposed to explain how the disordered structure of the Smad binding region facilitates extensive contact with the MH2 domain. In the model, the hydrophobic contact with each of the patches occurs with low affinity, whereas their combination achieves high affinity as well as specific binding (108). Consistently, these Smad-binding proteins partly share binding epitopes on Smad2/3, as suggested by binding competition experiments (109).

Recent X-ray crystallographic analyses support this model (110, 111, 112). There are six hydrophobic patches on the surface of the R-Smad MH2 domain that are available for interaction with binding proteins (110). Patches A1 to A3 are formed in a bundle region consisting of three α-helixes whose structures are altered by trimer formation, whereas patches B1 to B3 are located in a β-sandwich region whose structures are minimally affected by trimer formation (47, 111). Proteins that interact with the Smad2/3 MH2 domains are in contact with multiple patches in different combinations, thus increasing the binding specificity (Fig. 2*C*). For example, FoxH1 interacts with Smad2 *via* three patches, A2, B2, and B3 (110). The same hydrophobic patch (A1) is used by SARA (113, 114) in the monomeric MH2 domain before C-terminal phosphorylation and by c-Ski (110, 114) or CBP (112) after trimeric complex formation. These binding sequences do not share any amino acid sequence motifs or structural elements, suggesting that different binding proteins interact with the same patch in different ways, some using an amphipathic α-helix and others using β-strands. Consequently, SARA utilizes three patches (A1, B1, and B2), c-Ski uses two patches (A1 and A3), and CBP uses one patch (A1 only). This binding mode is effective because the three proteins share the same patch as part of the binding site, but they interact with Smad2/3 under distinct conditions: SARA interacts with unphosphorylated Smad2/3, whereas both c-Ski and CBP interact with Smad2/3 in a trimeric active complex, but they are mutually exclusive in binding to R-Smads (93, 115); these interactions have opposite effects on Smad complexes, as c-Ski is a corepressor and CBP is a coactivator. The amino acid sequences of patches A1, A3, B2, and B3 are well conserved among R-Smad proteins, whereas those of patches A2 and B1 are not (111). Therefore, binding to A2 and B1 dictates the selectivity of the interaction with TGF-β–activated Smads or BMP-activated Smads. Thus far, this model has been applied to limited examples, and further structural analyses need to be performed; for example, the usage of hydrophobic patches by transcription factors was resolved only for FoxH1.

Chimeric protein approaches were used to identify potential epitopes for several binding proteins. Smad1/2 chimeric proteins showed that Smad2 α-helix 2 (Gln-364–Tyr-366, corresponding to residues Gln-322–Tyr-324 in Smad3) is involved in the interaction with the SIM of the transcription factors Mixer, Milk, and FoxH1 (36, 116). The c-Ski–binding region of Smad3 was identified as Ser-266–Glu-267 and Gln-252–Thr-256 using Smad1/3 chimeric proteins (117, 118). Another approach based on point mutation of surface-exposed residues indicated that the Tyr297Ala mutation abolished interaction with SARA and c-Ski, although transcriptional activity was maintained (118). These potential binding epitopes do not match the FoxH1 or c-Ski binding region identified by crystallographic studies (110, 114). Such discrepancies remain to be explained.

#### Smad2/3–Smad cofactor interaction: *in vivo* context

**Figure 3:**
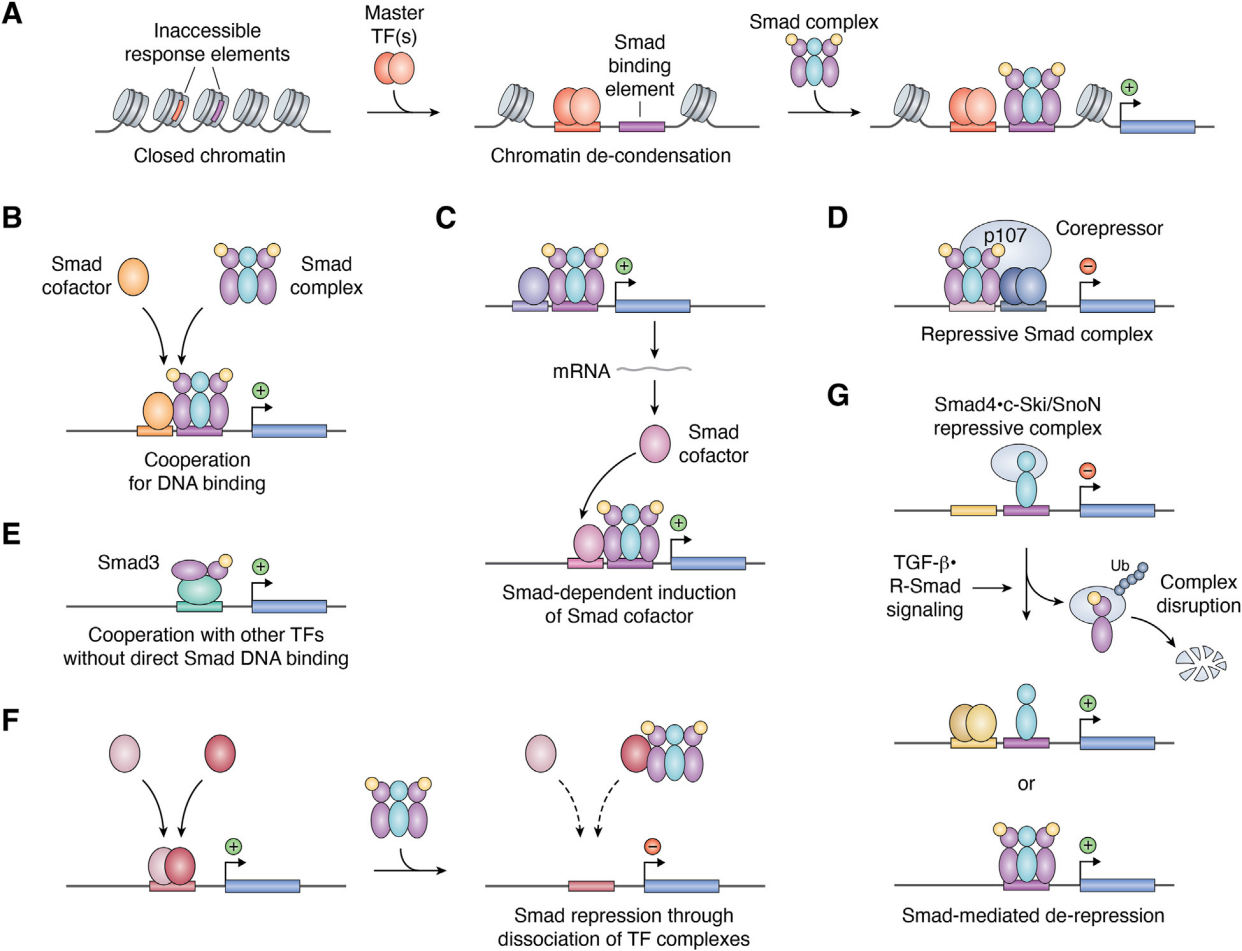
**Multiple modes of Smad2/3-dependent transcriptional regulation.***A*, master transcription factors initiate chromatin de-condensation and promote accessibility to surrounding regions, including a Smad-binding element, thus recruiting the Smad complex (119). *B*, the activated Smad complex and Smad cofactors cooperate in DNA binding (119). *C*, Certain Smad cofactors are induced by Smad-dependent transcription (self-enabling mechanism) (127, 128). *D*, repressive Smad complex containing E2F4 and p107 (145, 146, 147). *E*, Activated Smad3 can cooperate with transcription factors without its own DNA binding (35, 163, 164, 165). *F*, Smad proteins dissociate the heterodimeric complex of transcription factors, thus repressing gene expression (126, 169). *G*, the Smad4–c-Ski/SnoN repressive complex is disrupted by TGF-β/R-Smad signaling, thus activating gene expression (170, 171, 172). TF, transcription factor.

The cooperation between Smad proteins and Smad cofactors has been examined by biochemical as well as cell biological techniques, and many Smad-binding transcription factors have been identified (Table 1). Widespread usage of ChIP-seq or ChIP-chip analyses led to the identification of direct target genes of Smad proteins and revealed the co-presence of other transcription factor binding motifs. This facilitated the identification of novel transcription factors that cooperate with Smad proteins with deduced biological functions. Smad proteins occupy different enhancer regions of the same gene in a cell type–dependent manner (80, 81). Certain Smad cofactors bind to the genome prior to Smad binding and cooperate in gene regulation (Fig. 3*A*), whereas others cooperatively bind to the genomic DNA (Fig. 3*B*) (119).

**Table 1:** Transcription factors that cooperate with Smad2/3

Name	Smad selectivity	Binding domain	Signal -dependent interaction	Induction by TGF-β	Examples of target genes/proteins	*Gene SYMBOL* (target genes)	Cell response	Ref
ATF1	Smad2-specific	n.d.	n.d.	Yes	granzyme B, interferon-γ	*GZMB, IFNG*	immunosuppression in cytotoxic T cells	(150)
ATF2	Smad3/4	MH1	n.d.	Yes	IL-23a	*IL23A*		(324, 325)
ATF3	Smad3-specific	MH2	constitutive	Yes	inhibitor of DNA binding 1	*ID1*	stress response	(127)
ATOH8	Smad3-specific	n.d.	n.d.	n.d.	cyclin E2, cyclin dependent kinase 1	*CCNE2, CDK1*	inhibition of cell growth	(326)
c-Jun	Smad3/4	MH1 (S3)	Yes	Yes				(34, 327)
CTCF	Smad3-specific	MH1	n.d.	n.d.				(328)
E2F4	Smad3-specific	MH2	constitutive	n.d.	c-Myc	*MYC*	inhibition of cell growth	(145, 146, 147)
Ets1	Smad2/3/4	n.d.	constitutive	Yes	p21, PTHrP	*CDKN1A, PTHLH*	inhibition of cell growth	(80, 329, 330, 331)
ETV4	Smad2-specific	n.d.	Yes	n.d.	regulator of cell cycle (RGCC)	*RGCC*	smooth muscle cell differentiation	(332)
FoxH1	Smad2/Smad3/Smad4	MH2	Yes	n.d.	Mix.2	*Mix.2*	embryonic differentiation	(161, 162, 267, 333)
FoxL2	Smad3-specific	MH2	Yes	n.d.	follistatin	*FST*	follicle-stimulating hormone synthesis	(165)
FoxO1, 3, 4	Smad3/4	MH1	Yes	n.d.	p21	*CDKN1A*	inhibition of cell growth	(136, 137)
GATA-3	Smad3-specific	MH1	n.d.	n.d.	IL-10	*IL10*	immune suppression	(163)
GATA-4	Smad2/Smad3/Smad4	MH2	n.d.	n.d.	intestinal fatty acid binding protein	*FABP2*	gut epithelial gene expression	(334)
Gli2	Smad2/Smad3	n.d.	Yes	Yes	PTHrP	*PTHLH*	bone metastasis	(143)
HIF-1α	Smad3	MH1/MH2	Yes	n.d.	VEGFA	*VEGFA*	angiogenesis	(335)
HMGA2	Smad2/3/4	MH1/MH2 (S3)	Yes	Yes	Snail	*SNAI1*	induction of EMT	(336)
HNF4	Smad3/4	MH1 (S3)	Yes	n.d.	apolipoprotein			(337)
JunB	Smad3/4	n.d.	Yes	Yes	Wnt-7b	*WNT7B*	promotion of cell invasion	(128, 327)
KLF5	Smad2/3/4	n.d.	constitutive	n.d.	p15	*CDKN2B*		(338)
LEF1	Smad3	MH1/MH2	Yes	n.d.	*Xenopus* homeobox gene twin	*Xtwn*		(138)
MYOD1	Smad3	n.d.	n.d.	n.d	adenosine A1 receptor	*ADORA1*	myoblast differentiation	(125)
NFAT	Smad3	n.d.	n.d.	n.d.	forkhead box P3	*FOXP3*	T_reg_ cell differentiation	(291)
NFκB (p52)	Smad3	n.d.	Yes	n.d.	JunB	*JUNB*		(339)
Notch ICD	Smad3	MH2	Yes	n.d.				(164)
Oct4	Smad2/3	n.d.	n.d.	n.d.	left-right determination Factor 1, Nanog	*Lefty1, Nanog*	maintenance of cell stemness	(125, 340)
Olig1	Smad3	MH2	n.d.	Yes	PAI-1	*SERPINE1*	enhancement of cell motility	(216)
p53	Smad2/3	MH2	constitutive	No	p21, PAI-1	*CDKN1A, SERPINE1*	inhibition of cell growth	(141, 142)
p63	Smad2/3	n.d.	Yes	n.d.	Jun, Fos, laminin subunit beta 3	*JUN, FOS, LAMB3*	promotion of cell invasion	(341)
PU.1	Smad3	n.d.	n.d.	n.d.	immunoglobulin lambda like polypeptide 1	*IGLL1*	B cell differentiation	(125)
RREB1	Smad3	n.d.	Yes	No	Snail	*SNAI1*	induction of EMT	(140)
RORγt	Smad2/3	n.d.	Yes	n.d.	IL-17	*IL17A*	T_H_17 cell differentiation	(249)
RUNX3	Smad3/4	n.d.	n.d.	n.d.	p21	*CDKN1A*	inhibition of cell growth	(342)
Snail	Smad3/4	n.d.	constitutive	Yes	CAR, occludin, E-cadherin	*Cxadr, Ocln, Cdh1*	induction of EMT	(343)
Sox4	Smad2/3	MH2 (S3)	constitutive	Yes	N-cadherin, stromelysin-2	*CDH2, MMP10*	induction of EMT	(344)
Sox9	Smad2/3	MH2	Yes	n.d.	α1 (II) collagen	*COL2A1*	chondrogenesis	(166)
Sp1	Smad2/3	MH1	Yes	n.d.	p21	*CDKN1A*		(35)
TCF7L2	Smad3	MH2	Yes	n.d.	TMEPAI	*PMEPA*	negative feedback regulation	(345)
TFE3	Smad3	MH1	Yes	n.d.	PAI-1, Smad7	*SERPINE1, SMAD7*		(346, 347)
ZEB1	Smad2/3	n.d.	Yes	Yes			inhibition of cell growth	(348)
ZEB2 (SIP1)	Smad2/3	MH2 (S2)	Yes	Yes	*Xenopus* Brachyury	*Xbra2*		(348, 349)
ZIC3	Smad2	n.d.	n.d.	n.d.	Wnt-3	*Wnt3*		(350)
ZNF165	Smad3/Smad4	n.d.	Yes	n.d.	GTP-binding protein RAD	*RRAD*	cell growth and survival of triple negative breast cancer	(351)

n.d., not determined.

The selection of Smad cofactors in specific cellular contexts could be affected by the expression and activation levels of cofactors because high-affinity Smad cofactors may be preferentially associated with activated Smad complexes. However, the affinities of various Smad cofactors for the activated Smad complexes remain largely unknown. Similarly, the expression levels of Smad proteins themselves can affect cellular responses induced by TGF-β. High expression levels of Smad can cause low-affinity Smad cofactors to be complexed with Smad proteins. If Smad expression is low, only high-affinity Smad cofactors can function in cooperation with Smad proteins. Consistently, a two-fold reduction of the Smad3 gene dosage preferentially attenuates certain cell responses induced by TGF-β (120). Dose-dependent alterations in the genome-wide distribution of Smad-binding sites in mESCs have also been reported (121). Smad3 is induced by TGF-β signaling (122) and downregulated by Ras or STAT3 signaling (122, 123). Regulation of Smad3 expression, either through transcriptional or post-translational processes, is also an important factor that modulates context-dependent Smad signaling.

#### Cooperation with pioneer factors

The binding of transcription factors to regulatory regions can be affected by accessibility, whether they are situated in heterochromatins or euchromatins. A subset of transcription factors termed “pioneer factors” bind to their cognate DNA motifs even in closed chromatins and initiate chromatin de-condensation of the surrounding region, thereby promoting local DNA accessibility (124). Therefore, the chromatin pattern determines the accessibility of the Smad complexes. Such pioneer factors are often developmentally regulated master transcription factors that specify and maintain cell identity, and some of them, such as Oct-4, MyoD, and PU.1, interact with Smad proteins (125). In the *Lefty* enhancer region, Smad3 and Oct4 reside in the same transcriptional complex. However, master transcription factors recruit Smad3 to co-occupation sites to some extent by inducing the open chromatin state; this is because Oct4 binding peaks and Smad3 binding peaks in the same genomic locus are not always present in close proximity. In addition, nucleosomes are depleted in co-occupied sites in the absence of TGF-β signaling. Therefore, master regulators are likely to associate with the sites prior to the interaction with Smad3, and they facilitate the binding of Smad proteins to proximal genomic DNA regions. Physical interactions might not be necessary for functional cooperation, although many pioneer factors physically interact with Smad proteins. Co-occupation does not always mean functional cooperation. Although co-occupation by Smad3 and MyoD during myogenic differentiation has been reported (125), MyoD function is inhibited by physical interaction with Smad3 (126).

#### Signaling-regulated Smad cofactors

Some Smad cofactors are transcriptionally induced by TGF-β and function in the delayed phase of the transcriptional response (self-enabling mechanism (127), Fig. 3*C*). c-Jun and JunB are target genes of TGF-β and strikingly induced by TGF-β stimulation. They form homo- or heterodimers with c-Fos and bind to the activator protein 1 (AP-1) motif, also known as TPA-responsive element, to regulate gene expression. They can function as Smad cofactors. The AP-1 motif is often concentrated in Smad2/3 binding genomic regions (80, 128, 129, 130) or TGF-β–induced accessible chromatin regions (131). Jun family transcription factors are involved in the TGF-β–induced invasive properties of breast cancer cells (132): JunB cooperates with Smad3 to induce *WNT7B* and enhance tumor invasiveness (128). Ets1 is induced by TGF-β (133) and functions as a Smad cofactor, regulating a wide variety of target genes (80).

Smad cofactors that negatively regulate gene expression are also induced. TGF-β upregulates Id1 and Id3 in the early phase but represses them in the delayed phase in epithelial cells (127). This change is mediated by ATF3, which is induced by TGF-β. ATF3 is recruited to Smad complexes and represses Id1/Id3 gene expression. Target gene selectivity is ensured by the DNA binding properties of ATF3, which binds to cAMP response elements. This is an example of a self-enabling mechanism that mediates the time-dependent switch from an activator complex to a repressive complex. However, in glioma-initiating cell–like cells, TGF-β does not induce ATF3, and the TGF-β–induced expression of Id1 is maintained, playing a crucial role in gliomagenesis (134).

Smad cofactors can be induced by signaling pathways other than the Smad pathway. For example, ATF3 is induced by stress signaling, including activation of p38 MAPK, and cooperates with Smad3 (127, 135). In such cases, the signaling output is considered to be a result of signaling cross-talk. Co-activation of certain signaling pathways can alter the Smad–cofactor binding genomic regions, thereby affecting the gene expression program regulated by TGF-β. PI3K signaling induces inhibitory phosphorylation of FoxO transcription factors. FoxO factors are required for p21^WAF^ induction by TGF-β in keratinocytes (136, 137). Wnt-dependent induction of Smad–TCF complexes is involved in the expression of target genes such as the *Xenopus* homeobox gene *twin* (138). Ras signaling also modulates TGF-β signaling. For example, TGF-β–mediated induction of Snail, a core EMT-inducing transcription factor, is Ras activation dependent (139). RREB1 is activated by RAS–ERK signaling through the phosphorylation of the N-terminal region, which allows association with genomic DNA prior to the activated Smad complex, thus regulating the transcription of target genes including Snail (140). RREB1 plays a role in EMT in *KRAS*-mutant cancer cells and is also involved in embryonic development and adult fibrogenesis.

14-3-3ζ is a phosphoserine/threonine binding scaffold protein that is often overexpressed in breast cancer cells. It sequesters Yes-associated protein (YAP), a downstream effector of the Hippo signaling pathway, in a phosphorylation-dependent manner. Overexpression of 14-3-3ζ inhibits YAP activity, which indirectly destabilizes the p53 protein. p53 is a Smad cofactor involved in TGF-β–induced cytostasis (141, 142). Therefore, 14-3-3ζ can inhibit the cytostatic effect of TGF-β. In addition, 14-3-3ζ protects Gli2 from degradation by the ubiquitin–proteasome system. Gli2 is also a Smad cofactor, and in this case, it is involved in TGF-β–induced bone metastasis by upregulating parathyroid hormone–related protein (PTHrP) (143). Therefore, overexpression of 14-3-3ζ induces cofactor switching from tumor-suppressive to pro-metastatic (143). This is an example of how the context of Smad signaling can be altered by regulatory proteins.

### Repressed genes

TGF-β downregulates gene expression *via* various mechanisms, including destabilization of mRNAs or induction of miRNAs. In this section, we focus on the gene repression function in which the activated Smad complex directly affects the transcriptional machinery.

TGF-β inhibitory element (TIE) was first reported in the promoter region of stromelysin (currently known as matrix metalloproteinase 3, MMP-3) (144). The consensus sequence, 5′-GNNTTGtGa-3′, is also found in other TGF-β–repressed genes. Thereafter, Smad proteins were identified as signal-activated transcription factors, and their involvement in the regulation of transcription *via* TIE was extensively investigated in the c-*myc* promoter (145, 146, 147) because its repression is associated with TGF-β–induced cytostasis: downregulation of c-Myc, together with upregulation of cyclin-dependent kinase inhibitors, effectively suppresses cell growth. Because TGF-β inhibits cell cycle entry into S phase, many S-phase genes are indirectly downregulated by TGF-β in cell culture. This suppression is mediated by the Smad complex containing E2F4 and p107 (145, 146, 147), where p107 is a corepressor protein that regulates target gene downregulation (Fig. 3*D*). The E2F4–p107 gene-suppressing complex is formed after TGF-β stimulation, and the role of the E2F binding motif in TGF-β–induced gene repression was reported previously (148). This complex interacts with Smad3/4, but not with Smad2, to downregulate c-Myc. The repressive Smad binding site is located adjacent to the E2F4 binding motif in this element (147). A similar observation was made for a TIE in the promoter region of *PPARG*, which encodes peroxisome proliferator–activated receptor γ (149).

Repression of gene expression by Smad complexes does not necessarily require TIE. The Smad3–ATF3 cooperation described above is one such example. Another example is the cooperation of Smad with ATF1, which mediates the suppression of CD8^+^ cytotoxic effector function (150). TGF-β exerts immunomodulatory effects by downregulating genes associated with effector cells. ATF1 and CREB are induced by TGF-β and cooperate with Smad2/3 to interact with target gene promoters including Granzyme B, thereby repressing gene expression. Knockdown of both Smad2 and Smad3 inhibits the downregulation, whereas knockdown of Smad3 alone does not, suggesting that this downregulation is distinct from that mediated by TIE. Smad3 also directly recruits HDAC4 or HDAC5 to the Smad3–Runx2 complex, repressing osteocalcin gene expression during osteoblast differentiation (151). Similarly, HDAC8 is recruited to the Smad3–Smad4 complex and contributes to the downregulation of SIRT7 expression *via* histone H4 deacetylation (152). SIRT7 deacetylates Smad4 and promotes its degradation (153). Therefore, HDAC8 hyperactivates TGF-β signaling (152).

### Smad signaling in the absence of Smad4

Receptor-activated Smad2 and Smad3 form heteromeric complexes with Smad4 and translocate to the nucleus. Smad4 was previously thought to be an essential component of the functional Smad complex (98). Smad4 is often deleted in pancreatic and colorectal cancers, and loss of Smad4 was thought to severely compromise Smad signaling.

TGF-β, however, inhibits cell growth concomitant with the induction of p21^WAF^ in BxPC-3, a pancreatic cancer cell line lacking Smad4 (154). The activity of a p21^WAF^ promoter reporter construct was enhanced by overexpression of Smad2 or Smad3 in BxPC-3 cells, suggesting that Smad signaling occurs in the absence of Smad4 but not non-Smad signaling (154). TGF-β was also shown to enhance cell invasiveness in BxPC-3 cells (155). Recently, aggressive phenotypes induced by TGF-β in BxPC-3 cells were shown to be dependent on Smad2/3, demonstrating that Smad2/3 can transmit signals in the absence of Smad4 (156).

In Smad4-positive pancreatic cancer cells with KRAS activation, TGF-β induces apoptosis, thus suppressing tumor growth (157). It was suggested that induction of apoptosis, but not cytostasis, is a central mechanism of tumor suppression by TGF-β. The key downstream events are Smad4-dependent repression of KLF5 and Smad4-independent induction of Sox4. In Smad4-positive cancer cells, KLF5 is downregulated and Sox4, in the absence of KLF5, induces apoptosis by upregulating pro-apoptotic factors. However, in Smad4-negative cancer cells, KLF5 is not downregulated by TGF-β, and KLF5 and Sox4 cooperate to support tumor-initiating cells, thereby facilitating tumor progression. Smad4-independent genes include genes regulated by Smad2/3 complexes lacking Smad4 and genes regulated by non-Smad signaling pathways. Smad2/3 associates with the *SOX4* locus equally in the presence and absence of Smad4, suggesting that SOX4 is a Smad2/3-regulated gene. The composition of the Smad2/3 complex bound to the region, however, remains to be elucidated.

Smad2/3 are also known to regulate gene expression by forming complexes with proteins other than Smad4. TRIM33, a widely expressed nuclear protein, interacts with activated Smad2/3 and replaces Smad4 in the activated Smad complexes, thus regulating the expression of target genes distinct from those regulated by Smad2/3–Smad4 complexes: the Smad2/3–TRIM33 complex mediates erythroid differentiation, whereas the Smad2/3–Smad4 complex mediates cytostasis in hematopoietic progenitor cells (158). The Smad2/3–TRIM33 complex is more abundant in Smad4-deficient cells than in Smad4-positive cells, suggesting its involvement in Smad2/3 signaling in Smad4-deficient cells (158). Severe acute respiratory syndrome–associated coronavirus (SARS-CoV) nucleocapsid protein specifically interacts with Smad3 and promotes the association of Smad3 with p300 histone acetyltransferase while interfering with Smad3–Smad4 complex formation (159). Consequently, the SARS-CoV nucleocapsid protein attenuates TGF-β–induced apoptosis by suppressing the transcription of proapoptotic genes by the Smad3–Smad4 complex, whereas it supports the Smad4-independent Smad3 function in upregulating the expression of fibrotic genes in human peripheral lung epithelial cells (159). Thyroid transcription factor 1 (TTF-1, also known as NKX2-1) specifically interacts with Smad3 and inhibits Smad3–Smad4 complex formation, thereby suppressing target gene expression induced by TGF-β (82). TTF-1 recruits Smad3 to certain chromatin regions in the absence of TGF-β signaling, where Smad3 regulates a subset of target genes positively (*SDPR* and *FBP1*) or negatively (*LMO3*), independently of Smad4 (82). TTF-1 is a transcription factor that is expressed only in limited tissues including the lung, bronchi, thyroid, and forebrain. Therefore, it is involved in tissue-specific regulation of Smad signaling.

Smad4 was recently reported to be indispensable for BMP signaling, but not for Nodal signaling during embryogenesis in zebrafish (160). Inhibition of Smad2/3 signaling in the developing zebrafish embryo results in mesoendodermal defects, which is another example of robust Smad2/3 signaling in the absence of Smad4.

## Smad regulation of transcription that does not involve direct DNA binding

### Modulation of other transcription factors

Smad proteins can regulate gene expression without directly binding to DNA. During activation of the *Xenopus Mix.2* gene by the Smad2–Smad4–FoxH1 complex, FoxH1 represents a DNA-binding component of the transcriptional complex (161, 162). Similarly, Mixer and Milk cooperate with the Smad2–Smad4 complex in the activation of the *Xenopus goosecoid* gene through this mode of action (116).

Smad3–DNA binding is dispensable for the functional cooperation of Smad3 with Sp1 (35), GATA-3 (163), NICD (intracellular domain of Notch) (164), or FoxL2 (165) in transcriptional activation (Fig. 3*E*). Sp1, GATA-3, or NICD cooperates with a Smad3 Lys81Arg mutant that lacks DNA-binding activity, whereas FoxL2 cooperates with Smad3 lacking the MH1 domain. Smad4 is dispensable for the Sp1–Smad3 and GATA-3–Smad3 cooperativity. Other transcription factors that cooperate with Smad proteins without requiring DNA binding activity include Sox9 (166) and liver X receptor (LXR) (91). In these cases, Smad3 plays a role in recruiting coactivators, p300 for Sox9 and RAP250 and CBP for LXR, to the transcriptional complex.

### Smad-mediated repression of other transcription factors

TGF-β–induced gene repression sometimes accompanies Smad3-mediated inhibition of other transcription factors *via* physical interaction. During the process of myogenic or osteoblastic differentiation, Smad3 interacts with MyoD and prevents the formation of a functional MyoD–E12/47 complex that drives myogenic differentiation (126) (Fig. 3*F*). Smad3 also interacts with the myogenic factor MEF2C and interferes with its association with the coactivator protein GRIP-1, attenuating the expression of its target genes including *myogenin* (167). Similarly, Smad3 physically interacts with CBFA-1 (Runx2) and represses the expression of its target genes including *cbfa-1* and *osteocalcin*, resulting in inhibition of osteoblast differentiation (168). The repression is mesenchymal cell–specific and not observed in epithelial cells.

Polycyclic aromatic hydrocarbons (PAHs), including benzo[*a*]pyrene, are metabolically activated to be carcinogenic by cytochrome P450 1A1 (encoded by *CYP1A1*), which is transcriptionally induced by the aryl hydrocarbon receptor (AhR)/AhR nuclear translocator (ARNT) complex. TGF-β–activated Smad3 associates both with AhR and ARNT and inhibits induction of *CYP1A1* (169). Therefore, TGF-β suppresses the metabolic activation of PAHs by repressing *CYP1A1*, thus preventing carcinogenesis caused by PAHs.

### Smad-mediated de-repression

The transcription repressor function of the Smad4–c-Ski complex was first reported for the *SMAD7* promoter (170). The complex represses gene expression in the absence of TGF-β signaling input. Similarly, SnoN, a member of the c-Ski family, cooperates with Smad4 in the repression of the *SKIL* gene (encoding SnoN) (171). The repressive complexes are disrupted by TGF-β stimulation (172), which is possibly mediated by activated Smad2/3 (172) and RNF111 (also known as Arkadia) (173), an E3 ubiquitin ligase that targets c-Ski/SnoN (174, 175) (Fig. 3*G*). This regulatory system, which is considered one example of Smad2/3-mediated de-repression, plays an important role during T_H_17 cell differentiation.

Hypoxia-inducible factor 2α (HIF2α) regulates genes involved in hypoxic responses as well as intestinal iron absorption. Upon iron deficiency, *DMT1*, a gene encoding the iron transporter divalent metal transporter 1 for iron absorption, is induced in a HIF2α-dependent manner, whereas hypoxic HIF2α target genes are not. Thus, HIF2α regulates gene expression in response to specific environmental cues. Smad3 and Smad4 are both involved in this regulation, although Smad4 plays an essential role (176). In the resting state, Smad3 and Smad4 repress *DMT1* expression. Smad3 C-terminal phosphorylation is not required for the repression, whereas the DNA-binding ability of Smad4 is indispensable. Iron deficiency downregulates Smad3 and Smad4 *via* the ubiquitin–proteasome system, thereby de-repressing *DMT1* expression. Although the underlying mechanism remains to be elucidated, this appears to be a novel mode of transcriptional regulation by Smad proteins.

In cross-talk with the STAT3 system, TGF-β-activated Smad3 sequesters PIAS3, which inhibits STAT3 *via* physical interaction. Therefore, STAT3 is de-repressed to exert its transcriptional activity and induces Snail expression (177).

## Post-transcriptional RNA regulation pathways

In addition to transcriptional and epigenetic processes, the expression of protein-coding genes can be post-transcriptionally regulated by transcription factors at several steps, including pre-mRNA processing, mRNA export to the cytoplasm, translation of mRNA to proteins, and mRNA degradation (178). TGF-β–Smad signaling modulates these post-transcriptional processes *via* transcriptional regulation of downstream effectors. For example, TGF-β regulates mRNA splicing during epithelial–mesenchymal transition (EMT) by downregulating epithelial splicing regulatory proteins 1 and 2 (ESRP1 and 2) (179). However, Smad proteins, similar to other transcription factors (178), regulate gene expression by directly targeting RNA molecules. These findings are the result of a search for Smad-binding proteins using various methods, from the classical yeast two-hybrid screening to cutting-edge proteomics approaches, and several RNA-binding proteins have been identified as interacting partners of Smad proteins (180, 181, 182, 183, 184).

### MicroRNA processing

MicroRNAs (miRNAs) constitute a large family of small non-coding RNAs (20–26 nucleotides) that regulate gene expression *via* translational repression or by promoting the degradation of specific target mRNAs through association with the 3′-untranslated region (3′-UTR). They are processed from pri-miRNAs transcribed by RNA polymerase II to pre-miRNAs, and further processed to mature miRNAs. TGF-β signaling and BMP signaling regulate pri-miRNA expression positively or negatively, and also promote the processing of a subset of pri-miRNAs, including pri-miR-21 and pri-miR-199a, to pre-miRNAs by facilitating Drosha-mediated cleavage of pri-miRNAs (185). Recognition of target RNAs by Smad proteins is mediated by the 5′-CAGAC-3′ motif in double-stranded stem regions, through which the Drosha microprocessor complex is recruited to pri-miRNAs, thus facilitating processing to pre-miRNAs (186). This process does not require Smad4, and is thus considered a Smad4-independent process (185).

### mRNA splicing

In addition to the transcriptional regulation of splicing factors, activated Smad3 directly regulates mRNA splicing by forming a complex with phosphorylated poly (rC) binding protein 1 (PCBP1, also known as hnRNPE1), resulting in the displacement of the splicing machinery from PCBP1 (183). This mediates several processes, for example, exclusion of variable exons from CD44 transcripts, yielding the standard type CD44 isoform that is mainly expressed in mesenchymal cells (183), or exclusion of exon 12 from TGF-β–activated kinase (TAK1), yielding constitutively active TAK1, which promotes EMT and drug resistance (187). In the latter case, another RNA-binding protein, Rbfox2, is also required. Importantly, Smad3 has to be linker-phosphorylated at Thr-179 to interact with PCBP1 (183). Therefore, this process is regulated by Smad3 activation as well as linker phosphorylation through cross-talk signaling pathways (see below).

### m^6^A mRNA methylation

m^6^A methylation is a pre-mRNA modification that affects alternative splicing, translation, and mRNA stability (188). Smad2/3 signal-dependently interacts with the METTL3–METTL14–WTAP complex in human pluripotent stem cells (184), in which Smad2/3 are involved in the maintenance of pluripotent genes including *NANOG*. The METTL3–METTL14–WTAP complex mediates the co-transcriptional modification of nascent transcripts of Smad2/3-regulated genes with N^6^-methyladenosine (m^6^A) at the m^6^A-consensus motif, resulting in their destabilization. This mechanism ensures the timely shut-down of Smad2/3-regulated genes during the transition from naïve to primed pluripotent cells, thereby facilitating neuroectoderm differentiation. The regulation of Smad target gene expression by m^6^A methylation is also involved in modulating *JUN* (encoding c-Jun) and *JUNB* (encoding JunB) expression during EMT in lung cancer cells (189). c-Jun protein expression is regulated at the translational level, whereas JunB protein expression is regulated by controlling mRNA stability, and distinct reader proteins associated with the 3′-UTR of each mRNA. It remains unclear whether Smad3 recruits the METTL3–METTL14–WTAP complex to mRNAs encoding c-Jun or JunB, although this is likely because both genes are Smad3 target genes. However, whether this process requires the presence of Smad4 is not clear.

## Other regulatory functions of Smad2/3

Recent systemic proteomics approaches suggest that Smad2 and Smad3 have as-yet-unknown functions. Mass spectrometry analysis of Smad2 interacting proteins identified a mitochondria outer membrane G protein, mitofusin2 (MFN2), and Ras and Rab interactor 1 (RIN1) (190). Further analysis indicated that unphosphorylated Smad2 plays a role in mitochondrial fusion *via* these interactions. The MH1 domain binds to MFN2, whereas the MH2 domain recruits RIN1, a guanine nucleotide exchange protein that targets MFN2. Therefore, unphosphorylated Smad2 plays a role as a scaffold to facilitate mitochondrial fusion by activating MFN2. Accordingly, the shut-off of TGF-β signaling increases ATP production in a Smad2-dependent manner. This function is specific for unphosphorylated Smad2, and Smad3 cannot replace Smad2 in this function.

A link between Smad proteins and the DNA damage repair system has been suggested in several reports. In a proteomic approach, the ERCC1–XPF complex, an endonuclease essential for nucleotide excision repair, was shown to interact with Smad2/3, although the effect of the interaction on the activity of the ERCC1–XPF complex remains unclear (184). BRCA1, a well-known tumor suppressor gene product in breast and ovarian cancers, is involved in the repair of DNA double-strand breaks (191). Smad3 interacts with BRCA1 in response to TGF-β stimulation and inhibits BRCA1-dependent DNA repair in MCF7 breast cancer cells (luminal type) by disrupting BRCA1 nuclear complexes (192). Therefore, TGF-β enhances the cytotoxic effects of anti-cancer drugs that induce double-strand DNA breaks. However, these findings are largely based on ectopic overexpression experiments. It remains to be determined whether these findings are applicable to highly malignant breast cancer cells such as triple-negative breast cancer cells. C-terminally phosphorylated Smad2 colocalizes with proteins involved in DNA double-strand repair, such as 53BP1 and γH2AX, after ionizing radiation (193). This is a Smad2-specific event, and phosphorylation of Smad2 is inhibited by an inhibitor of ataxia telangiectasia mutated (ATM, a protein kinase), but not by a TGF-β type I receptor inhibitor. Many of these processes remain to be elucidated.

Protein kinase A (PKA) is a cytosolic enzyme composed of two regulatory subunits and two catalytic subunits, in which the regulatory subunits repress the catalytic subunits by physical interaction. Typically, cAMP associates with the regulatory subunit, altering its conformation to release the catalytic subunit, thus activating the kinase (194). TGF-β activates PKA by dissociating the catalytic subunit from the regulatory subunit independently of cAMP (195). Smad4 was found to interact with the regulatory subunit *via* its linker region (residues 290–300), which overlaps with SAD, through which Smad4 interacts with p300 (196). This interaction requires the presence of C-terminally phosphorylated Smad3, but not Smad2, accounting for the dependency on TGF-β stimulation (197). Therefore, activation of PKA by TGF-β is dependent on the activated Smad3–Smad4 complex. Notably, it is of early onset (activated from 15 min after stimulation) because it does not require transcriptional regulation. This pathway is involved in the phosphorylation of CREB and ATF1 (195), upregulation of fibronectin (195) and p21^WAF^ (197), and induction of an EMT phenotype (196).

## Regulation of Smad2/3 functions

### Posttranslational regulation

Regulation of Smad2/3 activity by acetylation, methylation, ADP-ribosylation, palmitoylation, or mono-ubiquitylation has been reported (198, 199, 200, 201, 202, 203, 204, 205). These post-translational modifications (listed in Table 2) enhance or attenuate Smad signaling by affecting stability, intracellular localization, interaction with binding partners or modifying enzymes, or competitive protection from other modifications. Here, we focus on the modification of Smad2/3 function by phosphorylation of the linker and other regions.

**Table 2:** Regulation of Smad2/3 by posttranslational modification

Modification	Site	Enzyme	Outcome	Ref
acetylation	Smad2 (K19)/Smad3 (K19)	p300/CBP (Smad2/3), P/CAF (Smad2)	enhancement of DNA binding	(198)
	Smad3 (K378)	p300/CBP	enhancement of Smad2/3 signaling	(199)
ADP-ribosylation	Smad3	PARP-1	inhibition of DNA binding	(200)
de-ADP-ribosylation	Smad3	PARG	(counteracting PARP-1)	(201)
methylation	Smad3 (K53/K333)	EZH2	required to interact with SARA	(202)
palmitoylation	Smad3 (C421)	ZDHHC19	enhancement of the association with the plasma membrane and activation of signaling	(203)
ubiquitylation (mono)	Smad3	(not specified)	inhibition of DNA binding	(204)
	Smad3 (T179 phosphorylated)	Smurf2	inhibition of trimer formation	(205)
ubiquitylation (poly)	Smad3	CHIP	degradation (independently of signaling)	(352)
	Smad2	ITCH	enhancement of Smad2 phosphorylation	(353)
	Smad2	NEDD4-2	degradation of activated Smad2	(354)
	Smad3 (K378)	pVHL	degradation	(355)
	Smad3	ROC1-SCFFbw1a	degradation of activated Smad3	(356)
	Smad2	Smurf2	degradation of activated Smad2	(357)
	Smad2	WWP1	degradation of activated Smad2	(358)
	Smad2/3	WWP2	degradation of unstimulated Smad2/3	(359)
deubiquitylation	Smad2/3	USP15	facilitation of DNA binding	(204)
	Smad3	USP7	facilitation of DNA binding	(360)
SUMOylation	Smad3	E3 SUMO-protein ligase PIAS4	inhibition of DNA binding	(361)
phosphorylation	Smad2 (Ser-465, Ser-467) Smad3 (Ser-423, Ser-425)	ALK-5, ALK-4, ALK-7	activation as a transcriptional regulator	(226, 227)
	Smad2 (Thr-252)	Araf	negative regulation (degradation)	(362)
	Smad2 (Ser-465, Ser-467)	ATM (inhibited by KU55933)	pSmad2 foci formation at DNA double strand break	(193)
	Smad2 (Ser-240)	CAM kinase	negative regulation	(363)
	Smad3 (Ser-418)	casein kinase 1γ2	negative regulation (degradation)	(364)
	Smad2 (Thr-8)	CDK2	negative regulation (inhibition of Smad2-Smad4 interaction)	(221)
	Smad3 (Thr-8, Thr-179, Ser-213)	CDK2/4	negative regulation of transcriptional activity	(220)
	Smad2 (Ser-255)	CDK4 (inhibited by palbociclib)	inhibition of the Smad complex to bind to the genome.	(365)
	Smad2 (Thr-220) Smad3 (Thr-179, Ser-208, Ser-213)	CDK8/9	negative regulation (degradation)	(208)
	Smad2 (Thr-220, Ser-245, Ser-250) Smad3 (Ser-208, Ser-213)	ERK	negative regulation (inhibition of nuclear localizaion of Smad2/3)	(206)
	Smad3 (Thr-179, Ser-204, Ser-208)	ERK	enhancement of ARE-dependent reporter activity	(366)
	Smad3 (Ser-204)	ERK	enhancement of *COL1A2* in mesenchymal cells	(367)
	Smad2 (Thr-197)	GRK2	negative regulation (inhibition of Smad2 C-terminal phosphorylation)	(368)
	Smad3 (Thr-66)	GSK-3β	negative regulation (degradation)	(369)
	Smad3 (Ser-204)	GSK-3β	negative regulation (inhibition of CBP binding)	(370)
	Smad2 (Ser-250, Ser-255) Smad3 (Ser-208, Ser-213)	JNK	enhancement of invasive capacity and negative regulation of *p15*^*ink4b*^ promoter activity	(209)
	Smad2 (Ser-245) Smad3 (Ser-204)	MPK38	enhancement of TGF-β signaling	(371)
	Smad2 (Ser-465, Ser-467) Smad3 (Ser-423, Ser-425)	Mps	not examined	(229)
	Smad2 (Ser-250) Smad3 (Ser-208)	NEMO-like kinase (NLK)	negative regulation (degradation)	(372)
	Smad2 (Thr-220) Smad3 (Thr-179)	OSR1 (oxidative stress-responsive kinase)	enhancement of Smad2/3 signaling	(373)
	Smad2 (Ser-417)	PAK2	negative regulation (inhibition of ALK5-Smad2 interaction)	(374)
	Smad2 (Ser-465)	PAK4	negative regulation (degradation)	(228)
	Smad2 (Ser-465, Ser-467) Smad3 (Ser-423, Ser-425)	PIM1	activation as a transcriptional regulator	(230)
	Smad3 (Ser-37, Ser-70)	Protein kinase C	inhibition of DNA binding	(375)
	Smad3 (Ser-309, Thr-388)	PKG	negative regulation (inhibition of nuclear translocation of phosphorylated Smad2/3)	(222)
	Smad3 (Ser-204, Ser-208)	ROCK (inhibited by Y27632)	up-regulation of p21^waf1^ protein, down-regulation of c-Myc protein	(376)
	Smad2 (Ser-465)	WNK1	negative regulation of Smad2 C-terminal phosphorylation	(377)
de-phosphorylation	Smad2 (Ser-465, Ser-467) Smad3 (Ser-423, Ser-425)	MTMR4	attenuation of TGFβ signaling by reducing the phosphorylation of R-Smads in early endosome	(52)
	Smad3 (Ser-423, Ser-425)	PP2A	attenuation of nuclear accumulation of TGF-β-induced Smad3	(378)
	Smad2 (Ser-465, Ser-467) Smad3 (Ser-423, Ser-425)	PP5	negative regulation of TGF-β signaling by decreasing the levels of Smad3 protein	(379)
	Smad2 (Ser-465, Ser-467) Smad3 (Ser-423, Ser-425)	PPM1A/PP2Cα	promotion of nuclear export of TGFβ-activated Smad2/3	(51)
	Smad2 (Ser-245, Ser-250, Ser-255)	SCP1/2/3	enhancement of TGFβ signaling	(218)
	Smad2 (Thr-8, Ser-245, Ser-250, Ser-255) Smas3 (Thr-8, Ser-204, Ser-208, Ser-213)	SCP1/2/3	increase of TGF-β-induced transcriptional activity	(219)
	Smad2 (Ser-465, Ser-467) Smad3 (Ser-423, Ser-425)	TNAP	negative regulation	(380)

**Figure 4:**
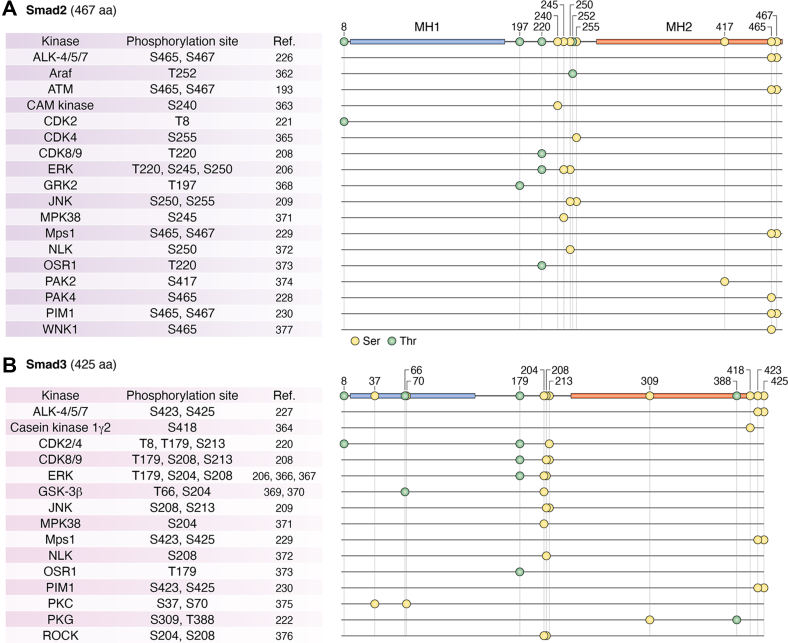
**Phosphorylation sites in Smad2 and Smad3.** Phosphorylation sites in Smad2 and Smad3 are shown, together with the responsible kinases and references. Some of the responsible kinases were determined on the basis of inhibitor studies (refer to Table 2). *A*, Smad2. *B*, Smad3. Schematic structures of Smad2 and Smad3 are presented on the top, in which the MH1 and MH2 domains are shown in *blue* and *red* bars, respectively. Abbreviations are as follows: ALK, activin receptor-like kinase; Araf, A-rapidly accelerated fibrosarcoma; ATM, ataxia-telangiectasia mutated; CAM, Ca^2+^/calmodulin-dependent protein kinase class of enzyme; CDK, cyclin-dependent kinase; ERK, extracellular signal–regulated kinase; GRK, G-protein–coupled receptor kinase; GSK, glycogen synthase kinase; JNK, c-Jun N-terminal kinase; MPK, murine protein serine–threonine kinase; Mps1, monopolar spindle 1; NLK, nemo-like kinase; OSR, oxidative stress–responsive kinase; PAK, p21-activated kinase; PIM, Proviral integration site for Moloney murine leukemia virus; PKC, protein kinase C; PKG, cGMP-dependent protein kinase or protein kinase G; ROCK, Rho-associated, coiled-coil containing protein kinase or Rho-associated protein kinase; WNK, With-No-Lysine (K) kinase.

The Smad2/3 linker region contains several serine/threonine residues that are phosphorylated by various serine–threonine kinases (Fig. 4, also listed in Table 2), upstream of which are tyrosine kinase receptors, G-protein–coupled receptors, and other signaling receptors. Therefore, it serves as a node for signaling cross-talk. The consequences of linker phosphorylation are signal-stimulatory or inhibitory, depending on the read-out of signaling. This is probably because functional activation during signal transmission is often linked to enhanced sensitivity to the inactivating machinery.

Oncogenic Ras or EGF stimulation, both of which suppress Smad-dependent reporter activity, was first shown to cause linker phosphorylation at four serine/threonine residues (206). TGF-β–induced linker phosphorylation was shown to require C-terminal phosphorylation (207) or complex formation with Smad4 (208), whereas hepatocyte growth factor (HGF)-induced linker phosphorylation is independent of C-terminal phosphorylation (209). Such a difference may reflect the usage of kinases upon different stimulations.

Smad2/3 are linker-phosphorylated in colorectal adenocarcinomas but not in normal colorectal epithelial cells (210). Smad3 phosphorylated at Ser-208/213 is located in the nucleus, whereas Smad2 phosphorylated at the corresponding sites (Ser-250/255) is detected in the cytoplasm in colorectal cancer specimens. These observations, together with cell-based assays, suggest that linker-phosphorylated, but not C-terminally phosphorylated Smad3, plays a role in transcriptional regulation (211).

Phosphorylation of Thr-179 in Smad3 forms a binding site for Pin1, a phosphorylation-dependent proline isomerase that recognizes Ser/Thr-Pro sites (212). Linker phosphorylation–resistant mutation of Smad3 attenuates TGF-β–induced cell motility and *in vivo* tumor growth and metastasis (213, 214, 215). Possible underlying mechanisms include alteration of the interaction with Smad cofactors such as Olig1 (216) or cooperation with PCBP in mRNA alternative splicing (described above) (183).

As mentioned above, functional activation is commonly associated with sensitivity to inactivating machinery. Thr-220 in Smad2 and Thr-179 in Smad3 are adjacent to the PY motif recognized by WW domain proteins. Thus, the phosphorylation of these sites creates a recognition mark for proteasomal degradation by serving as a docking site for ubiquitin ligases containing the WW domain. NEDD4-2 (NEDD4L) is thus able to interact with Smad2/3 after linker phosphorylation by CDK8/9, leading to ubiquitylation and proteasomal degradation (217). SCP phosphatases dephosphorylate the linker region and cancel the modulatory phosphorylation in the linker region (218, 219).

Phosphorylation of Thr-8 in Smad2/3 is mediated by CDK2 (220) and inhibits complex formation with Smad4, which might lead to the resistance of multiple myeloma cells to TGF-β–induced cytostasis or apoptosis (221). It remains unclear why phosphorylation of the MH1 domain affects the MH2 domain–mediated complex formation between Smad2/3 and Smad4. Ser-309 and Thr-388 of Smad3 are phosphorylation sites for cGMP-dependent protein kinase G (PKG) (222). Phosphorylation of these sites suppresses the nuclear translocation of Smad3, thus inhibiting TGF-β signaling. This mechanism is implicated in the suppression of cardiac fibrosis by atrial natriuretic peptide (ANP) (223), which transmits signals *via* the production of cGMP. Mutant studies of Smad3 Thr-388 phosphorylation show controversial results. Thr388Ala mutation causes resistance of Smad3 to the inhibitory effect of cGMP on signaling, exhibiting a positive effect on Smad3 signaling (224). By contrast, Thr388Val mutation results in decreased complex formation of Smad3– Smad4 or Smad3–CDK8 and subsequent failure to mediate the expression of fibrotic genes including α-smooth muscle actin, fibronectin, and collagen 1, thereby exhibiting a negative impact on Smad3 signaling (225).

Kinases that phosphorylate the C-terminal SSXS motif are not limited to type I receptors. Type I receptors phosphorylate Smad2 at Ser-465/467 and Smad3 at Ser-423/425 (226, 227), whereas p21-activated kinase 4 (PAK4) phosphorylates Smad2 at Ser-465 (228). PAK4-induced Smad2 phosphorylation does not activate Smad2, but it promotes ubiquitin-dependent degradation and is thus involved in negative regulation. Monopolar spindle 1, a mitotic kinase, phosphorylates the Smad2/3 SSXS motif and mediates nocodazole-induced Smad signaling (229). Similarly, PIM1 C-terminally phosphorylates Smad2/3 in clear cell renal cell carcinoma cells (230). Upon DNA double-strand break formation, ATM or its downstream kinase(s) phosphorylate Smad2 (193). The function of the resultant phospho-Smad2, which co-localizes with 53BP or γHATx, remains to be elucidated.

### Regulation of Smad2/3 via physical interaction

Many proteins regulate Smad2/3 function *via* physical interaction. Although we do not include the long list of these proteins here, we mention two examples of cross-talk with the PI3K signaling pathway and pattern recognition receptor signaling.

Akt physically interacts with unphosphorylated Smad3 and sequesters it in the cytoplasm, therefore inhibiting Smad3 phosphorylation and subsequent nuclear translocation (231, 232). Although this is a kinase activity–independent function of Akt, activation of Akt enhances the interaction; activation of the PI3K pathway can thus inhibit Smad signaling. This effect is Smad3-specific because Akt does not interact with Smad2 or Smad4.

IRF-3 is a transcription factor that is activated by virus infection *via* pattern recognition receptors such as RIG-I–like receptors and Toll-like receptors (2). Structural studies demonstrated the similarities between the C-terminal domain of IRF-3 and the MH2 domain of Smad proteins (233, 234) (Fig. 2*A*). Both IRF-3 and Smad proteins are activated by serine/threonine phosphorylation near or at the C-terminus. A closely related family protein, IRF-7, cooperates with Smad3 in the induction of interferon-β2 (235). By contrast, activated IRF-3 inhibits Smad3 signaling by forming a complex with unphosphorylated Smad3 to inhibit its activation (236). In addition, activated IRF-3 interferes with the interaction between Smad3 and coactivators, thus inhibiting Smad transcriptional complexes (236). Therefore, virus infection results in inhibition of iTreg differentiation by TGF-β, a well-designed cross-talk system for host defense.

### Long noncoding RNAs as regulators of Smad signaling

More than half of transcription factors interact with RNA through arginine-rich motifs, which facilitates the recruitment of transcription factors to chromatin (237). Smad3 was shown to interact with RNA through large internal loops or bulges with high affinity (238). A recent study identified Smad3 as an RNA-binding transcription factor despite showing low affinity for RNA (237). This suggests that, like other transcription factors, Smad-dependent transcription is regulated by RNA molecules. Certain long noncoding RNAs (lncRNAs) function in cooperation with Smad proteins in transcriptional activation. The lncRNA *DEANR1* recruits Smad2/3 to the *FOXA2* promoter, thereby promoting Smad2/3-induced FoxA2 expression to drive endodermal differentiation (239). The *DEANR1* gene is located downstream of *FOXA2*, and the *DEANR1* transcript localizes exclusively to the *FOXA2* gene locus, suggesting that the lncRNA *DEANR1* plays a limited role in recruiting Smad2/3. Similarly, the lncRNA *HAS2-AS*, which is induced by TGF-β, recruits Smad2/3 to the *HAS2* gene locus to induce its expression (240). Because HAS2 (hyaluronan synthase 2) is a crucial effector in the process of TGF-β–induced EMT, silencing of *HAS2-AS* attenuates EMT induced by TGF-β. In contrast to these *cis*-acting lncRNAs, the lncRNA *ELIT1* is induced by TGF-β and selectively associates with Smad3, thus recruiting activated Smad complexes to the promoter regions of various target genes including *SERPINE1*, *VIM, and ELIT-1* itself (241). Therefore, it behaves as a positive feedback regulator that contributes to sustained target gene expression. Similarly, *lincNMR* is induced by TGF-β and associates with Smad2 or Smad3 to promote the expression of *APOBEC3B*, which may contribute to tumor malignancy (242).

## Smad2 and Smad3: specificity and redundancy

Regulation of biological processes by Smad2/3 is in some cases dependent on both Smad2 and Smad3, whereas in other cases it is solely dependent on Smad2 or Smad3. Consistently, a gene expression profile resulting from Smad2 or Smad3 knockdown follows the same pattern (80). This raises a question regarding the molecular basis for Smad2-or Smad3-specificity, because the two proteins share 92% sequence similarity (25). At least two factors appear to be involved: distinct spatiotemporal distribution and differences in DNA binding or binding partner proteins.

Gene knockout studies indicate that *Smad2* knockout mice are embryonic lethal (E7.5–12.5) (243, 244, 245), whereas *Smad3* knockout mice are not (246, 247, 248), suggesting that Smad2 is indispensable for early embryonic development. This may be due, at least in part, to the specific spatiotemporal expression of *Smad2* and *Smad3* (*Smad*3 is not expressed in the extra-embryonic primitive endoderm), because mice in which *Smad3* cDNA was knocked into the *Smad2* locus are born and grow without apparent abnormality (29). Studies on *Smad2* conditional knockout mice revealed additional Smad2-specific functions *in vivo*, indicating that Smad2 is physiologically important for skin homeostasis, brain maintenance, and immune functions (249, 250, 251).

Three types of global *Smad3* knockout mice have been generated to date (exon 2 deletion (246), exon 8 deletion (247), and exon 1 deletion (248)), none of which exhibit embryonic lethality. However, *Smad3* knockout causes progressive abnormalities including impaired mucosal immune functions (247), bone abnormalities (252), emphysema associated with high expression of MMP-9 (253, 254), impaired skeletal muscle regeneration (255), and dopaminergic neurodegeneration (256). Positive effects have also been observed, including accelerated wound healing (257), protection from ionizing radiation–induced cutaneous injury (258), and protection from diabetic myocardiopathy (259). Detailed studies using conditional knockout mice revealed the complexity of the contribution of Smad3 to pathogenesis in different cell types (260, 261). In a myocardial infarction (MI) model, deletion of Smad3 in cardiomyocytes protects against MI by suppressing apoptosis and attenuating adverse remodeling (260), whereas its deletion in fibroblasts or macrophages promotes MI pathogenesis (260, 261). Therefore, Smad3 deletion in specific cell types is required to evaluate the functions of Smad3 *in vivo*. Although *Smad3* knockout mice are not embryonic lethal, Smad3 contributes to gene regulation during early embryonic development (262, 263). In *Smad2*^+/−^;S*mad*3^−/−^ mice, formation of the anterior visceral endoderm is normal, whereas that of the anterior axial mesendoderm is disrupted (262). Dose-dependent Smad2 and Smad3 signals cooperatively mediate cell fate decisions: high combinatorial Smad2/3 activity appears to be required for the latter process.

Smad2 phosphorylation is frequently used as a read-out for general Smad2/3 phosphorylation. This is probably because a low background pSmad2 antibody suitable for immunohistochemistry is commercially available. However, this can be confusing to readers because such studies are often conducted on Smad3-dependent processes. In the following sections, we describe how these functional specificities affect the pathophysiological context *in vitro* and *in vivo*.

### *In vitro* studies

The specific roles of Smad2 and Smad3 in cell responses have been explored using Smad2/Smad3 knockdown or knockout strategies. The effects of Smad proteins on cell responses vary according to the experimental setting. The Smad-dependency of cell responses or target gene regulation is cell-type dependent: induction of MMP-2 by TGF-β is Smad2-dependent in fibroblasts (264) while Smad3-dependent in PANC-1 pancreatic cancer cells (265). In fibroblasts, knockout of either *Smad2* or *Smad3* attenuates cytostasis induced by TGF-β (264). However, Smad3 predominantly mediates the cytostatic function of TGF-β in epithelial cells including HaCaT, Hep3B, SNU-368, and Huh7 cells (266). Knockdown of Smad2 in these cell lines enhances the cytostatic effect of TGF-β, suggesting that Smad2 antagonizes Smad3. The expression ratio of Smad2 to Smad3 may affect the signaling output. For experiments directly comparing Smad2 and Smad3 expression levels, an anti-Smad2/3 antibody with equal affinity to Smad2 and Smad3 (monoclonal, BD Biosciences) is quite useful (60).

The finding that *Smad2* knockout mice are embryonic lethal prompted studies examining Smad2-mediated transcriptional regulation during early development, especially the cooperation with FoxH1. Experiments in a cell culture system showed antagonism between Smad2 and Smad3 in the activation of the *goosecoid* gene: Smad2 promotes, whereas Smad3 suppresses the expression (267). Smad2 is specifically required for maintaining the pluripotency of primed pluripotent stem cells, *i.e.*, human embryonic stem cells and mouse epiblast stem cells, which also express Smad3 (268). Smad2 is involved in the induction of *Nanog* and the suppression of autocrine BMP signaling, which drive the differentiation of pluripotent cells toward mesoderm, trophectoderm, and germ cell lineage. To regulate *Nanog* expression, Smad2 binds to the *Nanog* promoter region in a stimulus-dependent manner, whereas Smad3 and Smad4 do not. Therefore, the transcriptional complexes regulating *Nanog* expression could be devoid of Smad3 and Smad4. Whether Smad2 exists as monomer, dimer, or trimer in the transcriptional complexes remains unknown. However, DNA binding transcription factor(s) should be included in the complex for its correct recruitment to target gene promoters.

TGF-β–responsive reporters that are solely dependent on Smad2 have not been developed to date. Although ARE-Lux with FoxH1 co-expression is often used as a Smad2-specific reporter (269, 270) based on the activity observed in *Smad2*^−/−^ mouse embryonic fibroblasts (264), it is activated by TGF-β in *SMAD2* knockout A549 human lung adenocarcinoma cells (271). The development of Smad2-specific reporters would help elucidate the differences between Smad2-and Smad3-specific gene regulation.

### Neurogenesis and brain functions

In neuronal cells, Smad2 is phosphorylated and localizes to the nucleus (272, 273, 274), suggesting that Smad2 plays important roles in neurogenesis and adult brain functions. Supporting this notion, central nervous system–specific *Smad2* knockout mice (*Nes*-*Cre*) exhibit behavioral abnormalities and severe cerebellar dysfunction associated with accelerated apoptotic cell death in the neo-cortex and the cerebellum, lack of migration and maturation of cerebellar granule cells, and defective dendritic differentiation of Purkinje cells with decreased synapse formation in the cerebellum (251). Due to the defects, these knockout mice cannot survive more than 3 weeks after birth. Smad2 is also indispensable for spatial learning memory and neuroplasticity in adult male mice, as expression of the *Smad2* gene is epigenetically regulated (the *Smad2* locus is demethylated by physical exercise) (270). Smad2 plays a role in the maturation of differentiating immature neuronal cells and in the plasticity of mature neurons (270). Smad2Δexon3 expression is higher in the brain of early postnatal mice than that of full-length Smad2 (272), whereas full-length Smad2 is the predominant form in peripheral tissues. This indicates that Smad2Δexon3 may play a specific role during neuronal development, which remains to be elucidated.

The roles of Smad3 in brain function have been elucidated using global *Smad3* knockout mice. *Smad3* knockout mice exhibit dopaminergic neurodegeneration and progressive α-synuclein aggregation in the brain. These are pathological phenotypes reminiscent of Parkinson’s disease (256, 275). Smad3 is also indispensable for long-term potentiation in the dentate gyrus, a process in which Smad3 appears to regulate GABA_A_ receptor-mediated neurotransmission (276, 277).

Smad2 and Smad3 function cooperatively in a complex regulatory process in developing neural tubes in chick embryos, in which overexpression of Smad3 or Smad2/3 promotes lateral migration of neural progenitors committed to differentiation and expression of pan-neural differentiation markers, whereas Smad2 overexpression has no effect (269). Consistently, the knockdown of Smad3 suppresses neurogenesis, indicating that Smad3 target genes are involved in promoting neurogenesis. Knockdown of Smad2, which was expected to have no effect, enhanced neurogenesis. This apparent discrepancy was explained by a model in which Smad2 target genes are activated by Smad2–Smad2–Smad4 complexes, whereas Smad3 target genes are activated by either Smad2–Smad3–Smad4 or Smad3–Smad3–Smad4 complexes. Alteration of the Smad2/Smad3 ratio leads to different cellular outputs in neural progenitor differentiation (269).

### Tumor progression

Because Smad2 mutations are found in various cancers although at a low frequency, Smad2 is thought to have tumor-suppressive functions (278). By contrast, the Smad3 mutation is rare and found mostly in colorectal cancer. This does not necessarily mean that Smad3 has no tumor suppressive effects, which are highly context-dependent (129). A dosage-dependent effect was reported for Smad3: two-fold reduction of the Smad3 gene dosage (based on the comparison between Smad3^+/+^ cells and Smad3^+/-^ cells) abrogates the pro-apoptotic response and attenuates the migratory response, but enhances the invasive response induced by TGF-β in immortalized mammary epithelial cells (120). Similarly, high levels of expression of Smad3 are required for its tumor suppressor function, whereas low expression of Smad3 is sufficient for its tumor-promoting function in mouse mammary epithelial cells (123). Constitutively activated Ras signaling results in the downregulation of Smad3 (123). Low levels of Smad3 expression are observed in human breast and gastric cancers (120, 279), and this decreased expression can change the effects of Smad3 from tumor suppressing to tumor promoting. Overall, however, the evidence supports that Smad2 suppresses, whereas Smad3 promotes tumor development, malignant progression, and metastasis in several types of cancer, although the underlying mechanisms are divergent. In this review, we focus on Smad2 function and its modulation by Smad3, which is often antagonistic to Smad2 in the context of tumor progression.

Because *Smad2* global knockout mice are embryonic lethal (243, 244, 245), *Smad2* heterozygous mice were analyzed for tumor formation (280). In contrast to *Smad4* heterozygous mice, which develop inflammatory polyps in the stomach and duodenum 1 year after birth (281), *Smad2* heterozygous mice do not develop gastrointestinal tumors (280). However, *Apc/Smad2 cis*-compound heterozygous mice show rapid malignant progression of intestinal tumors to invasive cancer compared with *Apc* single heterozygous mice (280). This suggests that Smad2 deletion itself is not tumorigenic, but accelerates the malignant progression of tumors, indicating that Smad2 has a tumor-suppressive function.

Keratinocyte-specific *Smad2* knockout mice show increased phorbol myristate acetate (PMA)-induced skin carcinogenesis (250). The underlying mechanism was shown to be de-repression of HGF expression associated with Smad2-mediated corepressor recruitment to the *Hgf* promoter (282). HGF thus induces tumor angiogenesis and promotes the progression of squamous cell carcinoma. HGF expression is enhanced by Smad3–Smad4 signaling. Therefore, imbalance of the Smad2/Smad3 ratio can have detrimental outcomes. A similar effect of Smad2 on tumor angiogenesis was reported in breast cancer cells, in which Smad2 suppressed the expression of vascular endothelial growth factor A, a crucial promoter of tumor angiogenesis (72). *Smad3* knockout mice are resistant to PMA-induced skin carcinogenesis, and this effect may involve the reduction of macrophage infiltration into tumor tissue in *Smad3* knockout mice (283).

In NRP-152 cells, which are non-tumorigenic prostate epithelial cells, suppression of TGF-β signaling induces malignant transformation (284). Knockdown of Smad2 alone is sufficient to transform the cells into tumorigenic cells in immunocompromised mice, whereas knockdown of Smad3 has no effect (285). However, the simultaneous knockdown of both Smad2 and Smad3 has a stronger effect on promoting *in vivo* tumor growth than the knockdown of Smad2 alone, suggesting that Smad3 plays a supporting role in the tumor suppressor function of Smad2. The reporter activities of 3TP-Lux and SBE4-Lux are also dependent on Smad2 in NRP-152 cells, in contrast to other cell lines such as NRP-154 tumorigenic prostate epithelial cells and A549 human non–small cell lung cancer cells, in which Smad3 plays a central role in inducing reporter activity (271, 284). Therefore, the function of R-Smads differs among cell lines, possibly depending on the cellular context. The molecular basis of this effect remains to be elucidated.

In A549 cells, Smad2 antagonizes the pro-metastatic function of Smad3 (286). A protein termed chaperonin containing TCP1 subunit 6A (CCT6A) selectively inhibits Smad2 function; therefore, upregulation of CCT6A or Smad2 mutation would trigger a pro-metastatic program mediated by Smad3. However, the data presented in this report are contradictory to the notion that Smad2 and Smad3 form a mixed trimeric complex: Smad2 and Smad3 are independently recruited to distinct genomic regions upon TGF-β stimulation. The loss of function of each protein does not affect occupancy of the other protein on the genome. This effect might be cell-type specific because Smad2 and Smad3 have redundant roles in transcriptional regulation in other cells (80, 287).

### Immune cell development

Excessive systemic inflammation was the first recognized phenotype in *TGF-β1*–knockout mice (288). Thereafter, many studies reported the involvement of TGF-β in immune cell differentiation, especially T cell subtype specification. Notably, TGF-β and programmed death ligand 1 (PD-L1) in the tumor microenvironment non-redundantly suppress tumor immunity. TGF-β also remodels the tumor environment to promote tumor progression. Therefore, a bifunctional protein in which the extracellular domain of TGF-β type II receptor (a TGF-β trap) is fused to anti-PD-L1 antibody is currently under evaluation in clinical trials to treat cancer (289). The important role of Smad3 in immune regulation was reported in 1999 on the basis of findings in global *Smad3* knockout mice, which exhibited impaired mucosal immunity and inflammatory responses (247, 257). Subsequently, conditional knockout studies revealed that Smad2 also plays a role in T cell differentiation (249, 287, 290).

T_reg_ cells are a subset of CD4^+^ T cells that negatively regulate immune functions to suppress auto-immune reactions and allergies. Differentiation from naïve CD4^+^ cells to T_reg_ cells is driven by the induction of a key transcription factor, FoxP3, by TGF-β. Two enhancer regions are affected by TGF-β (291, 292). Enhancer 1 is regulated by the cooperative action of Smad3 and NFAT, as indicated by the increase in reporter activity caused by overexpression of Smad3 but not Smad2 or Smad4 (291). Enhancer 2 is primarily regulated by the binding of STAT5, the activation of which is dependent on repression of the negative regulator SOCS3 by Smad3-mediated TGF-β signaling (292). These studies support the principal role of Smad3 in T_reg_ induction. However, *in vivo* studies using T cell–specific Smad2/3 knockout mice (using *CD4*-*Cre* or *Lck*-*Cre*) demonstrate that whereas single knockout of *Smad2* or *Smad3* partly attenuates this process, double knockout completely abrogates it (249, 287, 290). Therefore, Smad2 and Smad3 play essential but redundant roles in the induction of T_reg_ cells by TGF-β, although the role of Smad2 might not be mediated by cooperation with NFAT on the promoter but by indirect effects. Redundant roles of Smad2 and Smad3 are also observed in the suppression of T_H_1 differentiation by TGF-β (287).

T_H_17 cells are another subset of T cells that have pro-inflammatory functions mediated by IL-17 secretion. Smad2 and Smad3 are intricately involved in this process. The master regulator of T_H_17 differentiation is RORγt, encoded by *Rorc*. STAT3 activated by IL-21 drives *Rorc* expression. Unphosphorylated Smad3 represses *Rorc* expression by interacting with STAT3 and PIAS3, whereas linker-phosphorylated Smad2 (Ser-255) positively regulates it by interacting with STAT3 and p300 (293). RORγt expressed by the action of STAT3 cooperates with Smad2 and induces IL-17 expression (249), whereas it is repressed by physical interaction with Smad3 (290).

During *in vivo* induction of T_reg_ cells by *Clostridium* species in the colon, dendritic cells secrete TGF-β *via* auto-induction, and Smad3 plays a positive role, whereas Smad2 plays a negative role in the transcription of the *TGF-β1* gene (294).

### Fibrosis

TGF-β induces the conversion of resident fibroblasts to activated myofibroblasts and increases the production of α-smooth muscle actin and extracellular matrix proteins including fibronectin and collagen, whereas it suppresses their degradation, thereby promoting fibrosis (295). TGF-β also induces the expression of lysyl oxidases that cross-link and stabilize collagen (296), as well as cytokines and growth factors that promote fibrosis including connective tissue growth factor (CTGF) (297, 298). TGF-β signaling in epithelial, endothelial, or immune cells is also involved in the fibrotic process according to experiments using *TβRII* conditional knockout mice (*Ksp*-*Cre*;*TβRII*^flox/flox^ (299), *Tie2*-*Cre*;*TβRII*^flox/+^ (300), and *LysM*-*Cre*;*TβRII*^flox/flox^ (301)). A mouse model of *Schistosoma mansoni* infection–induced liver fibrosis is exceptionally known to be independent of TGF-β signaling (302).

This signaling pathway is principally mediated by Smad3: *Smad3* knockout mice show attenuated fibrotic responses to various stimuli, and this is associated with suppressed mRNA expression of type I procollagen. Mouse models of tissue fibrosis include pulmonary fibrosis (253, 303, 304), renal fibrosis (305, 306), hepatic fibrosis (307, 308), skin fibrosis (309), colorectal fibrosis (310), cardiac fibrosis (311, 312, 313), and peritoneal fibrosis (314). Autoinduction of TGF-β mediated by Smad3 is important for the fibrotic process, as suggested by high expression levels of TGF-β1 and pSmad2 in the pulmonary fibrotic region, which is not observed in the lungs of *Smad3* knockout mice (304).

In a model in which Smad2 or Smad3 was deleted in activated fibroblasts expressing the *periostin* gene, Smad2 deletion had no effect on fibrosis induced by transaortic constriction, whereas Smad3 deletion attenuated it (315). By contrast, Smad2 deletion in kidney tubular epithelial cells increases renal fibrosis induced by unilateral ureteral obstruction (316). Similarly, Smad2 deletion in the peritoneum exacerbates peritoneal fibrosis induced by continuous ambulatory peritoneal dialysis, indicating that Smad2 plays a suppressive role (314). In the same model, systemic deletion of *Smad3* attenuated peritoneal fibrosis. Although studies on *Smad2* conditional knockout mice have not been performed extensively, Smad3 appears to play a primary role in transmitting signals for fibrosis.

## Future perspectives

Smad proteins were originally identified as signaling-activated transcription factors. The basic framework of their signal transmission was established soon thereafter (4), and the unique features of Smad signaling were unveiled (3). However, there are still many unanswered questions. Smad proteins form activated trimeric complexes with multiple combinations of R-Smads and Co-Smad that behave as a transcriptional half unit. The mechanism by which these distinct complexes function to achieve fine regulation of gene expression is not well understood. The molecular architecture of the transcriptional machinery containing the activated Smad complex also remains unknown. Although Smad4 was considered to be an essential component of the Smad complex in the early days after its identification, the role of activated Smad complexes devoid of Smad4 was recently reported (156, 157, 158, 159). Although the functional differences between Smad2 and Smad3, which have 92% sequence similarity, were demonstrated *in vivo* using conditional knockout mice, the underlying mechanisms remain to be elucidated. Smad proteins function in a dose-dependent manner: at high dosage, Smad3 is tumor suppressive, whereas at low dosage, it is pro-metastatic (120, 123). Therefore, simple loss-of-function experiments may not be sufficient to evaluate the functions of Smad proteins.

To date, hundreds of Smad binding proteins have been identified, but many of them are not sufficiently characterized to reflect their functional relationship with Smad proteins. Those identified in the early days have primarily been characterized using an overexpression system only, and loss-of-function experiments have not been performed. Some may be interaction partners that are not relevant to physiological processes, whereas others may be involved in unknown important processes. Smad proteins are activated as transcriptional regulators upon C-terminal phosphorylation. Therefore, the functions of Smad proteins have been conventionally analyzed with the input of TGF-β signaling. However, Smad proteins that are not C-terminally phosphorylated were recently shown to play roles in the regulation of gene expression. The recruitment of unphosphorylated Smad3 by FoxH1 to target gene promoters (263) or the corepressor function of unphosphorylated Smad3 in the presence of STAT3 and PIAS3 (293) has been reported. Unphosphorylated STAT proteins are also involved in transcriptional control (317). The role of unphosphorylated Smad2 as a scaffold protein in the regulation of mitochondrial function is also intriguing. Smad7, an inhibitory Smad that lacks the C-terminal SSXS motif and is therefore not C-terminally phosphorylated, also functions as a scaffold protein in the activation of p38 MAPK (318). Inspiration drawn from a deep understanding of biological processes, as well as state-of-art biochemical analyses, will help identify novel modes of Smad-dependent regulation of pathophysiological processes.

*In situ* detection of Smad signaling is frequently based on the analysis of phospho-Smad2. However, such methods have limitations because of the presence of nuclear antagonists that act downstream of Smad phosphorylation events. These are c-Ski (93, 94), SnoN (95), Evi1 (96), Mel1 (97), and TGIF (92). In addition, virus-derived inhibitors of Smad signaling generally target the activated Smad complexes (319, 320, 321, 322, 323). Therefore, detection of phospho-Smad proteins simply indicates the existence of signaling input, but does not demonstrate responses to the signaling. Novel *in situ* detection systems to monitor Smad signaling would facilitate research on the pathophysiological roles of Smad proteins.

Because Smad3 is thought to be involved in pathogenic processes including tissue fibrosis and tumor progression, several so-called “Smad3-specific inhibitors” have been developed. However, evaluation of their specificity mostly relies on the selective inhibition of Smad3 phosphorylation. A widely used inhibitor, SIS3, was recently shown to target transcriptional processes downstream of Smad phosphorylation and inhibit TGF-β–induced reporter activities even in *SMAD3* knockout cells (271); therefore, its specificity for Smad3 is not warranted. A biochemical platform to precisely evaluate Smad3-dependent signaling should be used. Notably, previously unknown phenotypes of *Smad3* knockout mice have been unveiled; for example, global *Smad3* knockout mice show dopaminergic neurodegeneration reminiscent of Parkinson’s disease (256, 275). Therefore, systemic inhibition of Smad3 should be carefully conducted. One possible way to overcome this limitation is to target specific Smad cofactors. Smad cofactors interacting through the MH2 domain appear to share hydrophobic patches (110). Therefore, blocking one hydrophobic patch might simultaneously affect the interaction of many Smad cofactors. Elucidating the molecular architecture of the transcriptional machinery containing the activated Smad complex might provide useful information for developing strategies to target Smad-dependent transcription. An alternative method is to inhibit Smad3 signaling in a cell type–specific manner. Deletion of Smad3 in different cell types showed opposite effects in a myocardial infarction model (260, 261). Thus, cell-type specific inhibition of Smad3 signaling may be more efficient. For this, conditional knockout studies in different cell types present in the pathogenic microenvironment, as well as the application of drug-delivery systems to specific cell types, are required, which might be a challenge for the next decade.

## Data availability

All data are contained in the manuscript.

## Conflicts of interest

The authors declare no conflicts of interest with the content of this article.
